# Metabolomic serum abnormalities in dogs with hepatopathies

**DOI:** 10.1038/s41598-022-09056-5

**Published:** 2022-03-29

**Authors:** Carolin A. Imbery, Frank Dieterle, Claudia Ottka, Corinna Weber, Götz Schlotterbeck, Elisabeth Müller, Hannes Lohi, Urs Giger

**Affiliations:** 1 Vetsuisse Faculty, University of Zürich, 8057 Zürich, Switzerland; 2 Laboklin GmbH & Co. KG, 97688 Bad Kissingen, Germany; 3 Institute for Chemistry and Bioanalytics, School of Life Sciences, University of Applied Sciences Northwestern Switzerland, 4132 Muttenz, Switzerland; 4 PetMeta Labs Oy, 00300 Helsinki, Finland; 5 University of Helsinki and Folkhälsan Research Center, 00250 Helsinki, Finland; 6 Section of Medical Genetics, University of Pennsylvania, Philadelphia, PA 19104 USA

**Keywords:** Metabolomics, Hepatology

## Abstract

Hepatopathies can cause major metabolic abnormalities in humans and animals. This study examined differences in serum metabolomic parameters and patterns in left-over serum samples from dogs with either congenital portosystemic shunts (cPSS, n = 24) or high serum liver enzyme activities (HLEA, n = 25) compared to control dogs (n = 64). A validated targeted proton nuclear magnetic resonance spectroscopy platform was used to assess 123 parameters. Principal component analysis of the serum metabolome demonstrated distinct clustering among individuals in each group, with the cluster of HLEA being broader compared to the other groups, presumably due to the wider spectrum of hepatic diseases represented in these samples. While younger and older adult control dogs had very similar metabolomic patterns and clusters, there were changes in many metabolites in the hepatopathy groups. Higher phenylalanine and tyrosine concentrations, lower branched-chained amino acids (BCAAs) concentrations, and altered fatty acid parameters were seen in cPSS dogs compared to controls. In contrast, dogs with HLEA had increased concentrations of BCAAs, phenylalanine, and various lipoproteins. Machine learning based solely on the metabolomics data showed excellent group classification, potentially identifying a novel tool to differentiate hepatopathies. The observed changes in metabolic parameters could provide invaluable insight into the pathophysiology, diagnosis, and prognosis of hepatopathies.

## Introduction

The liver plays a central role in the metabolism of proteins, carbohydrates, lipids, fatty acids, and amino acids involved in both anabolic and catabolic processes. Therefore, both primary liver and systemic diseases that secondarily affect the liver can result in major metabolic abnormalities that impact quality of life and life expectancy^1,2^. Various congenital hepatic diseases, such as inborn errors of metabolism and structural anomalies, are recognized in humans^3,4^ and dogs^5^. Congenital portosystemic shunts (cPSS) are commonly seen vascular liver anomalies in young dogs^6^ and cats^7^, but occur very rarely in children^8,9^. In addition, there are many acquired acute and chronic hepatic diseases in humans and animals related to infectious, inflammatory, degenerative, vascular, neoplastic, drug- or toxin-related processes, as well as hepatopathies of idiopathic origin, which are associated with high serum liver enzyme activities^10–13^. Of major human public health concerns are acute and chronic hepatitis virus A to E infections and alcohol-related liver diseases^14^, which are not recognized in dogs^15^. However, viral hepatopathy in dogs can be induced by canine hepatitis adenovirus type 1, although this is now exceedingly rare due to general vaccination strategies^16^. While the majority of hepatitides in dogs are classified as idiopathic^11^, bacterial, parasitic, toxic, and immune-mediated causes are also recognized^17,18^.

A variety of diagnostic screening tests are applied to clinically detect hepatopathies and their consequences in human and veterinary medicine, including serum hepatic enzyme activities and liver function parameters, followed by liver imaging, biopsy, and other specific tests^2,12^. While methods to assess certain classes of specific metabolites in health and disease have been used for more than half a century, techniques that permit more comprehensive analyses of biological samples with metabolomic analyses have only become available in this millennium. Advanced technologies such as nuclear magnetic resonance (NMR) spectroscopy and mass spectrometry (e.g., gas chromatography-mass spectrometry [MS], liquid chromatography-MS) permit the precise identification and quantification of large numbers of metabolites, thereby informing on the impact of physiological and pathological processes on the metabolome and metabolic pathways^19,20^. There are several studies in human patients indicating characteristic metabolic abnormalities in patients with different hepatopathies^21,22^.

Metabolomic investigations of hepatopathies in veterinary medicine are still very limited^23,24^. Only a few studies have evaluated serum metabolomics with specific MS methods in dogs with liver diseases^25,26^. Moreover, determining of distinct groups of metabolites such as amino acids in dogs with cPSS has been reported^27^. Here, we utilize proton (^1^H) NMR spectroscopy to assess and compare the serum metabolome of dogs with cPSS, dogs with high serum liver enzyme activities (HLEA), and dogs with blood test results in the normal reference intervals. Our specific goals were to determine, if (1) specific metabolic abnormalities could be detected in serum from dogs with different hepatopathies by ^1^H NMR spectroscopy, (2) serum metabolomic patterns differ between the two hepatopathies as well as compared to control dogs, and (3) machine learning models would be capable of correctly differentiating groups solely based upon metabolomics data.

## Methods

### Samples and groups

Left-over serum samples submitted for routine testing to a veterinary diagnostic laboratory (Laboklin GmbH & Co. KG, Bad Kissingen, Germany) between September 2020 and June 2021 from dogs with cPSS, HLEA, and apparently healthy younger (≤ 3 years [yr]) and older adult (> 3 yr) dogs with blood test results within the reference intervals were included in this study.

For sample selection, the laboratory’s electronic database was searched for:

- cPSS group: samples with high serum bile acid (BA) concentrations (> 40 µmol/L, reference interval 0–15 µmol/L) and no or only slightly elevated age-related serum liver enzyme activities from dogs ≤ 3 years of age^28^.
- HLEA group: samples with markedly increased serum alanine transaminase (ALT) activity (> 450 U/L, reference interval 0–55 U/L, representative of a moderate to marked increase^29^) without laboratory evidence of other diseases.
- Control groups: samples with serum chemistry panel and complete blood count (CBC) results in the reference intervals (age-dependent^28^) were selected for control dogs ≤ 3 yr (Controls ≤ 3 yr) and > 3 yr of age (Controls > 3 yr), which were later combined because of only very minor metabolomic differences between them.

Available information on breed, age, sex, neutering status, and other data received from submission forms, the medical consult service, as well as blood test results were gathered and reviewed. For all groups, dogs were excluded if there was information available on concurrent diseases (e.g., infectious diseases, hyperadrenocorticism, cancer, or others) based upon submission forms and other laboratory tests performed at Laboklin. Furthermore, attending veterinary clinicians who submitted serum samples from dogs with high serum BA were contacted, and additional clinical information (including clinical signs and/or abdominal imaging or surgery) was obtained by Laboklin’s medical consult service (C.A.I.) to support the diagnosis of cPSS and exclude other diseases based upon the provided clinical information.

The laboratory’s inventory of frozen samples was screened for left-over serum samples with a residual volume of ≥ 300 µL. These serum samples were originally submitted to the laboratory after centrifugation and removal of clotted blood and were delivered at room temperature with a transport time of ≤ 1 day (except two samples which were kept and arrived chilled within two days). Samples with observed hemolysis, lipemia, and/or severe icterus were excluded. Frozen serum samples were thawed, aliquoted (1.8 ml CryoPure tubes, Sarstedt AG & Co. KG, Nürnbrecht, Germany) and refrozen at -80 °C until shipment for metabolomic analysis within ≤ 8 months. Serum chemistry analyses (Cobas 8000 c701 analyzer, Roche Diagnostics, Mannheim, Germany) were performed on thawed samples, if not already undertaken during routine testing. Results of CBC (ADVIA 2120i, Siemens Healthcare GmbH, Erlangen, Germany or Sysmex XT2000i, Sysmex Deutschland GmbH, Norderstedt, Germany) were reviewed if available.

### Serum metabolomic analyses

Serum samples were shipped frozen overnight on ice packs to PetMeta Labs Oy, Helsinki, Finland. Metabolomic analysis was conducted using a targeted ^1^H NMR method optimized and validated for canine serum and plasma^30^. A similar method has been described and largely used for human serum and plasma^31,32^. Details of the method are published elsewhere ^30–32^. Briefly, the highly automated method utilizes a Bruker AVANCE III HD 500 MHz spectrometer equipped with a 5 mm triple-channel (^1^H, ^13^C, ^15^N) z-gradient Prodigy probe head and a cooled high-throughput sample changer SampleJet (Bruker Corp., Billerica, Massachusetts, USA). NMR analyses were conducted at Nightingale Health Oyj, Helsinki, Finland. Submitted samples were lightly mixed and centrifuged to remove any potential precipitates^33^. Each sample was transferred into a NMR tube (Bruker Corp., Billerica, Massachusetts, USA) and carefully mixed with sodium phosphate buffer in an automated process using a JANUS Automated Workstation equipped with an 8-tip dispense arm with Varispan (PerkinElmer Inc., Waltham, Massachusetts, USA)^31^. Two NMR spectra (so called LIPO and LMWM windows) were acquired automatically with standardized parameters from each sample. The LIPO window shows a conventional water-suppressed ^1^H NMR spectrum with broad and overlapping resonances arising mainly from proteins, lipoproteins, and lipids^33^. The LMWM window suppresses the broad signals of most macromolecules and lipids with T_2_-relaxation-filtering, improving the detection of low-molecular-weight metabolites^33^. The acquired NMR spectra are automatically processed using in-house scripts optimized for canine samples^30^, including background control, baseline removal, and signal alignments^31^. Absolute concentrations of the metabolic measures were obtained with a proprietary software with integrated quality control^32^. The quantification was based on regression modeling^32^ and results in the quantification of 123 measurands, including detailed lipoprotein analyses with lipoprotein subclass concentrations and compositions, amino acid concentrations and several amino acid ratios, fatty acids in both molar and relative units, and concentration of triglycerides, cholesterols, glycolysis-related metabolites, albumin, and creatinine^30^.

### Statistical analyses

#### Univariate analyses

Univariate statistical analysis was performed using the SPSS Statistics (version 26; IBM Corp., Armonk, New York, USA) and MS Office Excel (Microsoft Corp., Redmond, Washington, USA) software programs. All continuous data were assessed for normal distribution. Differences in age, routine serum clinical chemistry results, and metabolomics data were evaluated using a Kruskal–Wallis test^34^, and for serum BA concentrations using a Mann–Whitney U test^35^. Kruskal–Wallis tests for metabolomic analyses were adjusted with Bonferroni correction^36^. Sex and neutering status between groups were evaluated using chi-square tests. The level of significance was set at *p* < 0.05.

#### Multivariate analysis

Concentrations of metabolomics data missing at random were imputed by the median of the corresponding variable, whereas concentrations below the detection limit were imputed with a zero value. Principal component analysis (PCA), partial least squares-discriminant analysis (PLS-DA), the hierarchical cluster heatmap, and hierarchical cluster analysis (HCA) of the serum metabolomics data were performed using MetaboAnalyst 5.0^37^ using auto-scaled variables. A hierarchical cluster heatmap with samples and parameters clustered was created to visualize changes in the most discriminative parameters identified by variable importance in projection (VIP) scores in PLS-DA (selection of top 20 variables). Hierarchical cluster analyses were performed using the Ward clustering algorithm and the Euclidean distance measure^38^. Machine learning methods were performed with WEKA 3.95^39^. Models applied here were simple logistic regression^40^, support vector machines^41^, k-nearest neighbors (KNN) algorithm^42^, Multilayer Perceptron (MLP) Classifier^43^, Random Forest^44^, and multinomial naïve Bayes^45^. The default settings of the parameters for the respective WEKA implementation were used for all machine learning methods. Machine learning models were tested using 10-fold full cross-validation for each model.

Briefly, PCA is an unsupervised pattern recognition method that is used for dimension reduction. The first two principal components explain the most possible variance of the data. Scores plots derived by PCA reveal clustering or separation of individual data points (samples), thereby enabling determination of similarities^46^. In contrast, PLS-DA is a supervised statistical analysis that uses prior knowledge of group assignments and thereby maximizes the separation of groups. Partial least squares-discriminant analysis is based on PLS-regression using a dummy variable encoding the class membership^47^. Hierarchical cluster analysis determines sample clustering by merging samples into homogeneous clusters based on homogeneity of measured serum metabolites.

Univariate analysis and PCA of the serum metabolomics data revealed only minor differences between the younger and older adult Control groups. Thus, both Control groups were combined in the further statistical analyses of the serum metabolomics data in this study to simplify analyses and increase the statistical power for machine learning approaches.

### Ethics declaration

The use of left-over blood samples for research purposes is stated in the general terms and conditions of Laboklin GmbH & Co. KG and was approved by the government in Würzburg, Lower Franconia, Bavaria, Germany (RUF-55.2.2-2532-1-86-5).

## Results

### Samples, demographics, and routine laboratory test results

The serum samples selected fulfilled the respective group’s entry criteria for dogs with either high serum BA concentrations (cPSS) or high liver enzyme activities (HLEA) or originating from younger and older adult dogs with serum chemistry and CBC results within age-dependent reference intervals and thus no laboratory evidence of liver or other disease (Control groups) (Supplementary Table S1). While every effort was made to select cases based upon the laboratory and clinical information available, the animals were not examined by the authors.

**Figure 1:**
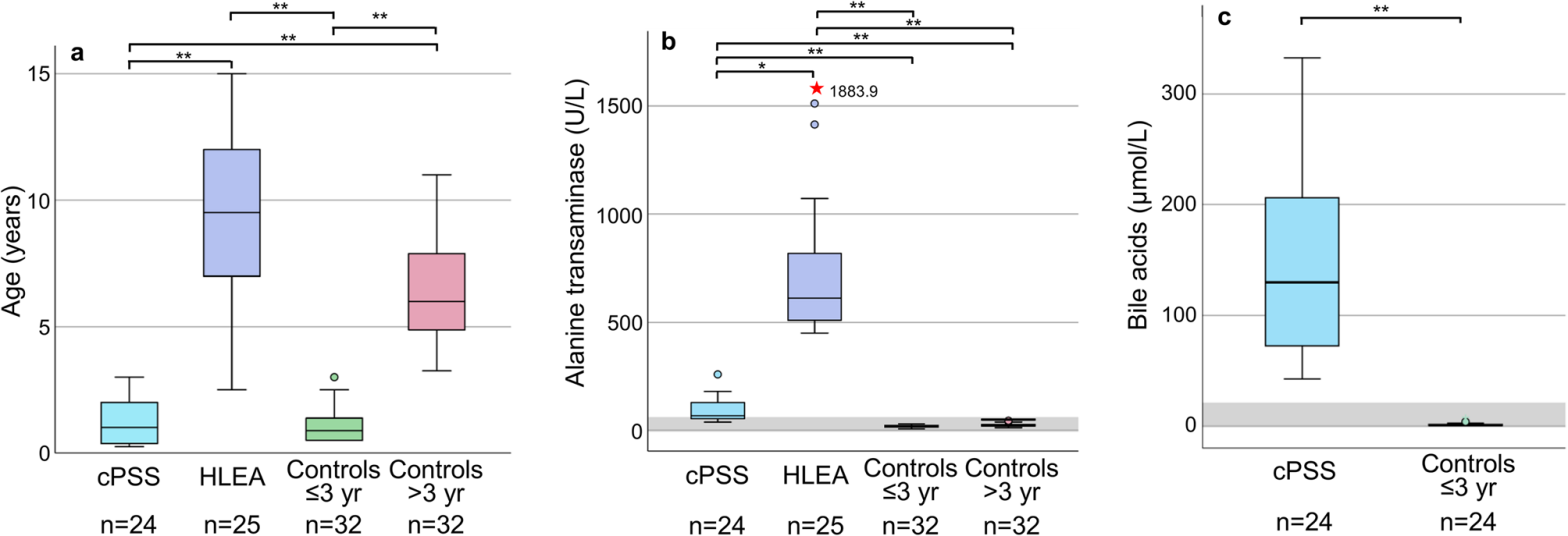
(**a**) Age, (**b**) alanine transaminase activities (ALT), and (**c**) bile acid (BA) concentrations in serum samples from dogs with cPSS (n = 24), HLEA (n = 25), and younger and older adult control dogs (n = 32, for each). Bile acid concentrations were determined in all dogs with cPSS and in 24 of 32 controls dogs of ≤ 3 yr. Boxes indicate the lower to upper quartile (25th–75th percentile), and median value. Whiskers extend to minimum and maximum values. Outliers are shown as individual open circles or red stars. Grey boxes indicate reference intervals. Lines above figures reflect significant differences between specific groups (*p < 0.05; **p < 0.001).

A total of 113 left-over canine serum samples were included and analyzed by ^1^H NMR spectroscopy in this study, including 24 cPSS serum samples and 25 HLEA serum samples. The Control groups of dogs ≤ 3 yr and > 3 yr included 32 serum samples each. Serum samples were mostly from Germany (95%) and rarely other European countries (Czech Republic, Italy, Luxembourg, and Sweden). As designed, dogs with cPSS and Controls ≤ 3 yr were of a similar age, as were the HLEA and Control group > 3 yr (Fig. 1a, Supplementary Table S1). Mixed breed dogs accounted for the largest proportion enrolled in any group. No breed predilection and no sex differences were observed between groups, except a higher number of neutered dogs with HLEA (Supplementary Table S1).

As determined by the inclusion criteria, dogs with HLEA showed significantly higher serum ALT activities than all other groups (Fig. 1b; Supplementary Table S1). Serum BA concentrations were highly elevated in dogs with cPSS compared to Controls ≤ 3 yr, which were only available for these two groups (Fig. 1c; Supplementary Table S1).

### Metabolomic analyses

The ^1^H NMR spectroscopy platform for metabolic profiling studies was recently validated for 123 metabolic parameters with 8,247 canine serum and plasma samples^30^. In the present study, all 105 serum metabolite concentrations were identifiable and measurable. Furthermore, 11 relative concentrations of fatty acids and seven selected amino acid ratios could be calculated, totaling 123 parameters as in the recently published validation study for canine samples^30^. Medians for all parameters for younger as well as older adult control dogs fell in the previously established serum reference intervals for dogs of all ages with 90% confidence intervals (CIs)^30^, except for the median concentrations of glutamine and L-VLDL-triglycerides, which were slightly lower in the Control group ≤ 3 yr. Moreover, for a few parameters, 25th or 75th percentiles of the Control groups in this study fell slightly below (concentrations of glutamine, citrate, acetate, glycoprotein acetyls [GlycA], and large very-low-density lipoprotein [L-VLDL]-triglycerides) or slightly above (concentration of glycine, glycine/branched-chain amino acid [BCAA] ratio, glycine/valine ratio, relative concentration of linoleic acid, relative concentration of omega-6 fatty acids, omega-6/omega-3 fatty acid ratio, high-density lipoprotein [HDL] particle size, and concentration of small low-density lipoprotein [S-LDL]-triglycerides) the published serum reference intervals for dogs of all ages^30^ (Supplementary Table S2).

**Figure 2:**
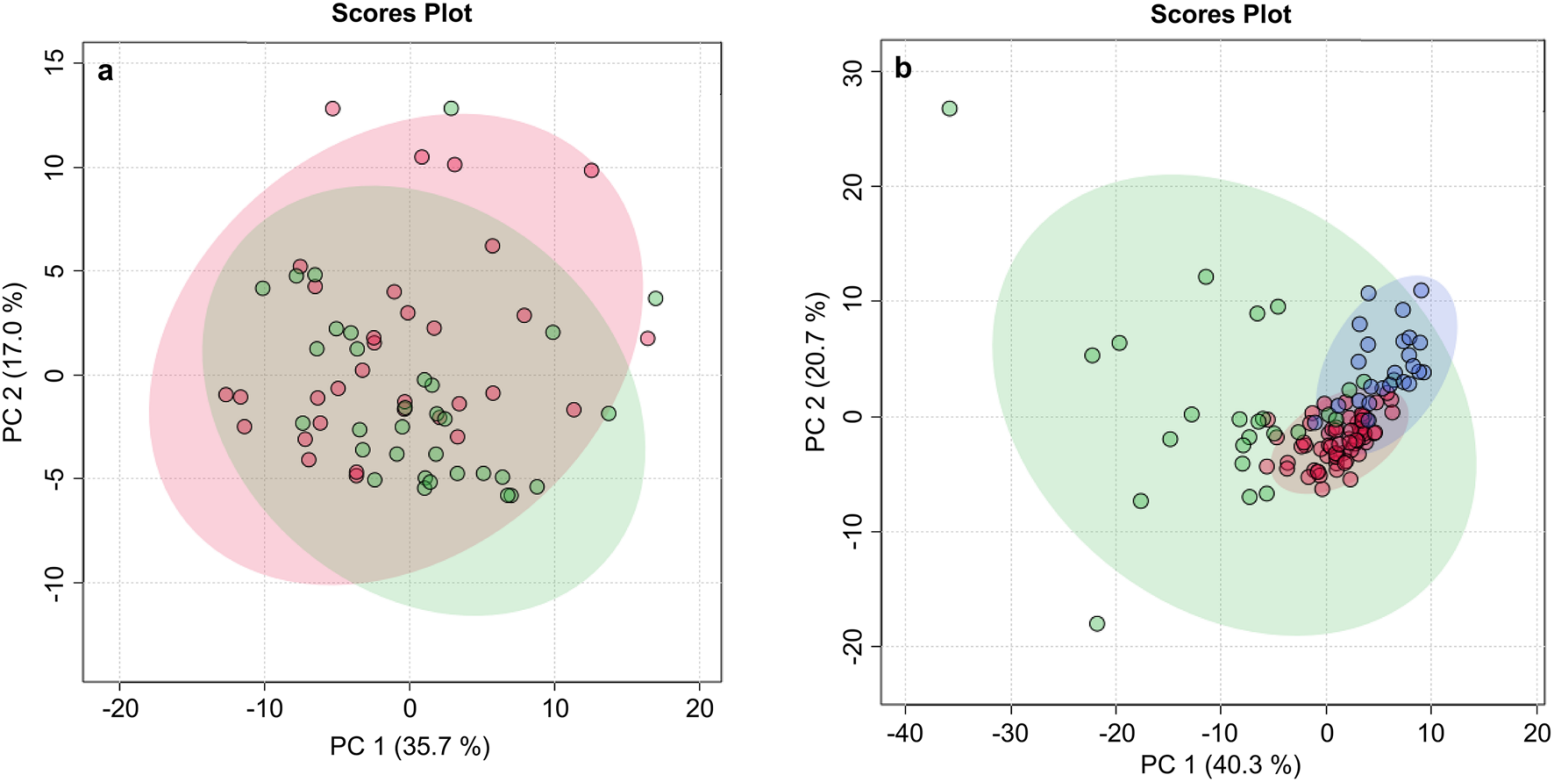
(**a**) Scores plot of principal component analysis (PCA) showing overlap between serum samples from dogs of the younger (≤ 3 yr, green) and older adult (> 3 yr, red) control dogs, indicating near-complete overlap (n = 32, for both). (**b**) Scores plot of PCA of the serum metabolome from dogs with cPSS (blue, n = 24) and HLEA (green, n = 25) and the combined Control group (red, n = 64). Shaded circles represent 95% confidence intervals, while colored dots illustrate individual samples. The axes are labelled by the first and second principal component (PC 1 and 2, respectively) with the percentages of variance of the data explained by that principal component in parentheses.

Because of the expected age difference of dogs with cPSS (younger) and HLEA (older), two age-dependent Control groups with dogs that were younger or older than 3 years of age were initially set up. However, univariate testing revealed only one slight difference between the two Control groups for citrate concentrations (*p* = 0.049), with the younger Control group having slightly lower median serum concentrations and the 25th percentile below the reference interval (Supplementary Table S2). Moreover, PCA demonstrated near-complete overlap between the clusters of the younger and older adult Control groups (Fig. 2a). Therefore, the two Control groups were combined in the subsequent statistical analyses of the metabolomics data.

Univariate testing of serum metabolomics data showed significant differences in 100 of 123 parameters between dogs with cPSS, HLEA, and/or the combined Control group (Supplementary Table S2).

**Figure 3:**
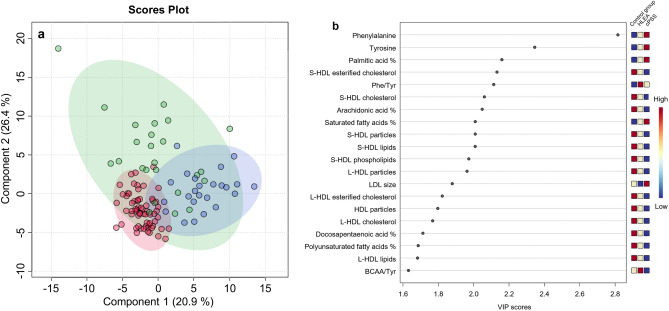
(**a**) Scores plot of partial least squares-discriminant analysis (PLS-DA) based on metabolomics data between serum samples of dogs with cPSS (blue, n = 24), HLEA (green, n = 25) and combined Controls (red, n = 64). Shaded circles represent 95% confidence intervals, while colored dots illustrate individual samples. The axes are labelled by the first and second components with the percentages of variance of the data explained by that component in parentheses. (**b**) Variable importance in projection (VIP) scores of component 1 of the PLS-DA identifies the top 20 discriminating parameters in descending order of importance from the serum metabolomics data of dogs with cPSS (n = 24), HLEA (n = 25) and the combined Control group (n = 64). The colored legend on the right indicates the relative abundance of variables, with red and blue indicating high and low values, respectively, while beige illustrates neutral values.

The PCA revealed clustering of canine samples from cPSS, HLEA, and combined Controls based on the serum metabolome (Fig. 2b). The total variance of the first two principal components contributed 61% in the PCA model for all three groups (PC 1 = 40.3%, PC 2 = 20.7%). Both the cPSS and the combined Control group showed tight clustering, while the HLEA group clustered more broadly, overlapping with the other two clusters. Many variables were found to influence the projection and separation of the groups as reflected in marginalized metabolites in the PCA’s loadings plot (Supplementary Fig. S1). To maximize the separation of the groups observed by PCA, PLS-DA was applied. In the PLS-DA model for the three groups, the first two components contributed to a total variance of 47.3%. The three groups showed robust clustering, which partially overlapped, particularly with the larger HLEA cluster (Fig. 3a). The PLS-DA model was validated by a 10-fold cross-validation with R^2^, Q^2^, and accuracy as displayed in Supplementary Fig. S2. All figures show a robust model with four components being selected as optimal number of components based on the Q^2^ criterion. Furthermore, a permutation test with 1000 permutations was performed. The results displayed in Supplementary Fig. S3 demonstrate that the model is not overfitting the data. None of the 1000 permutations achieved the same separation distance defined by the ratio of the between-group sum of the squares and the within-group sum of the squares (B/W-ratio)^48^. The variables most responsible for separation in the PLS-DA were ranked according to the VIP scores. The metabolites identified as most discriminating between the three groups were phenylalanine, tyrosine, S-HDL esterified cholesterol and S-HDL cholesterol concentrations, and relative concentrations of palmitic acid, arachidonic acid, and saturated fatty acids, followed by others (Fig. 3b).

**Figure 4:**
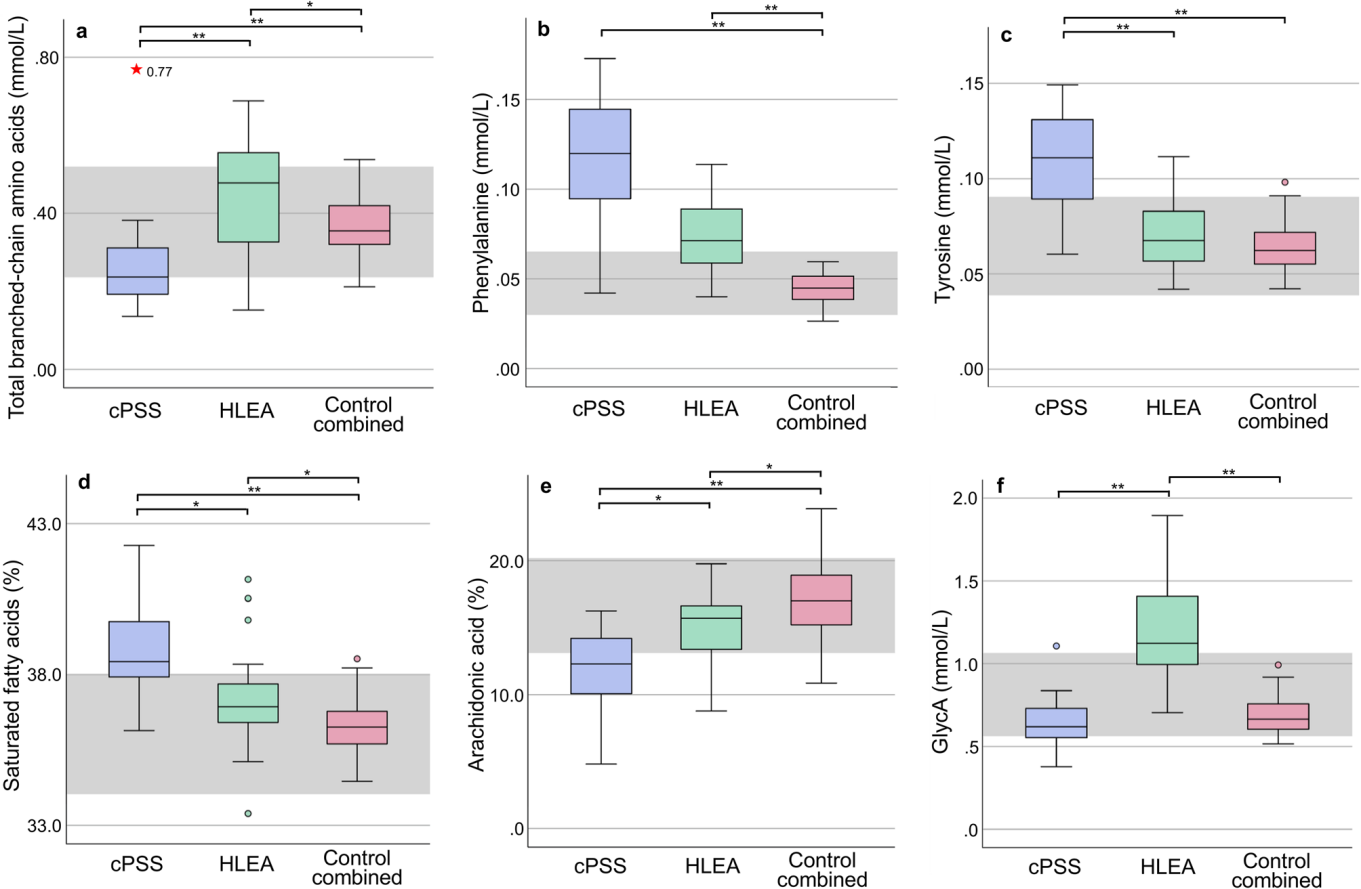
Concentrations (mmol/L) of total branched-chain amino acids (BCAAs) (**a**), phenylalanine (**b**), tyrosine (**c**), percentage of saturated fatty acids (**d**), percentage of arachidonic acid (**e**), and concentrations (mmol/L) of glycoprotein acetyls (GlycA) (**f**) in serum samples from dogs with cPSS (n = 24), HLEA (n = 25), and the combined Control group (n = 64). Boxes indicate the lower to upper quartile (25th–75th percentile) and median value. Whiskers extend to minimum and maximum values. Outliers are shown as individual open circles or red stars. Grey boxes indicate reference intervals. Lines above figures reflect significant differences between specific groups (*p < 0.05; **p < 0.001).

Among the altered metabolites between groups were multiple amino acids, with some having high discriminatory power. For instance, dogs with cPSS showed higher serum concentrations of the aromatic amino acids (AAAs) phenylalanine and tyrosine, as well as of histidine, compared to the Control group. Moreover, dogs with cPSS had significantly lower concentrations of total BCAAs, particularly leucine and valine (but not isoleucine), while dogs with HLEA had higher concentrations of total BCAAs, particularly isoleucine and valine (but not leucine) compared to the Control group (Fig. 4a, Supplementary Table S2). Phenylalanine and tyrosine were found to be relevant parameters for group separation in PLS-DA (Fig. 3b). Phenylalanine concentrations were elevated in dogs with both hepatopathies (Fig. 4b). While being a relevant marker in PLS-DA to distinguish between the three groups, there was no significant difference by univariate testing between dogs with cPSS or HLEA. However, serum tyrosine concentrations were only high in dogs with cPSS (Fig. 4c).

Several serum lipoprotein concentrations as well as the lipoprotein compositions were altered in dogs with cPSS and HLEA compared to the Control group (Supplementary Table S2, Supplementary Fig. S1). In particular, concentrations of small-HDL particles, lipids, cholesterol, esterified cholesterol, and phospholipids were found to have a discriminative power in PLS-DA (Fig. 3b) and were decreased in dogs with cPSS and, to a lesser extent, in the HLEA group. Similarly, serum concentrations of large-HDL particles, lipids, cholesterol, and esterified cholesterol were decreased in dogs with cPSS, but normal in the HLEA group (except large-HDL particles), and therefore were discriminatory parameters in PLS-DA (Fig. 3b). In contrast, dogs with HLEA showed increased concentrations of VLDL and LDL particles. Finally, total cholesterol concentrations were higher in dogs with HLEA, while total cholesterol concentrations were lower in dogs with cPSS compared to the Control group. Moreover, serum total triglyceride concentrations were trending to be increased in dogs with HLEA compared to the Control group (*p* > 0.05) (Supplementary Table S2).

Absolute fatty acid concentrations were generally decreased in serum from dogs with cPSS (except docosahexaenoic acid) and mostly increased in dogs with HLEA compared to the Control group (Supplementary Table S2). Moreover, the relative quantities of some serum fatty acids were altered in dogs of either disease state compared to the Control group. For instance, dogs with cPSS showed lower relative concentrations of polyunsaturated fatty acids, such as the omega-6-fatty acid arachidonic acid and docosapentaenoic acid, compared to the Control group (Supplementary Table S2). In contrast, the relative concentrations of saturated fatty acids (Fig. 4d), specifically palmitic acid, were increased in dogs with cPSS. Both relative concentrations of arachidonic and palmitic acid were identified as relevant parameters for group separation by PLS-DA (Fig. 3b). In contrast, dogs with HLEA had higher relative concentrations of stearic acid and decreased relative concentrations of arachidonic acid (Supplementary Table S2, Fig. 4e). The decrease in the relative concentration of arachidonic acid was more apparent in dogs with cPSS (Fig. 4e).

The inflammatory biomarker GlycA, measured by ^1^H NMR spectroscopy, was increased in dogs with HLEA compared to Controls, while dogs with cPSS had serum GlycA concentrations within the reference interval (Fig. 4f).

In addition, serum acetate and lactate concentrations were increased in both hepatopathies (Supplementary Table S2). Citrate concentrations were low and high in dogs with cPSS and HLEA, respectively. However, the citrate and lactate changes were marginal and mostly in the established reference interval^30^. Serum concentrations of glucose and pyruvate did not differ between the groups (Supplementary Table S2).

**Figure 5:**
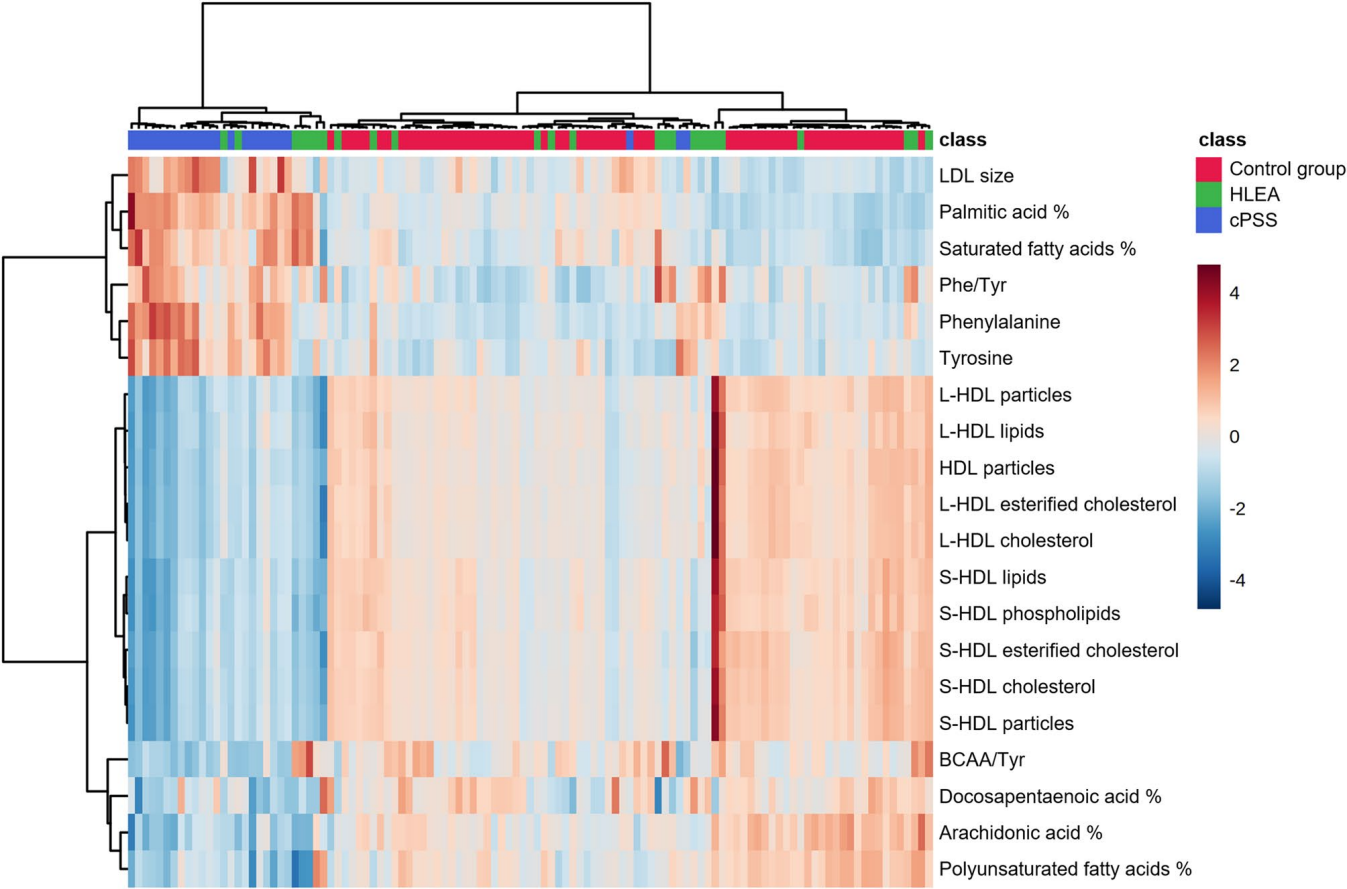
Hierarchical cluster heatmap (for samples and variables) of serum metabolomic results using the top 20 parameters identified by partial least squares-discriminant analysis (PLS-DA) variable importance in projection (VIP) scores. Each column represents one serum sample with group marking colored at the top: blue cPSS (n = 24), green HLEA (n = 25), and red Control group (n = 64). The colored legend on the right indicates the relative metabolite concentrations, with different red and blue intensities indicating high and low values, respectively. Horizontal and vertical black lines depict clustering of samples and parameters.

In a hierarchical cluster heatmap of the above-mentioned parameters identified by VIP scores in PLS-DA, the pattern and clustering for the cPSS samples was distinct, while the samples of dogs with HLEA and the Control group were intermixed and showed less intragroup homogeneity (Fig. 5).

**Figure 6:**
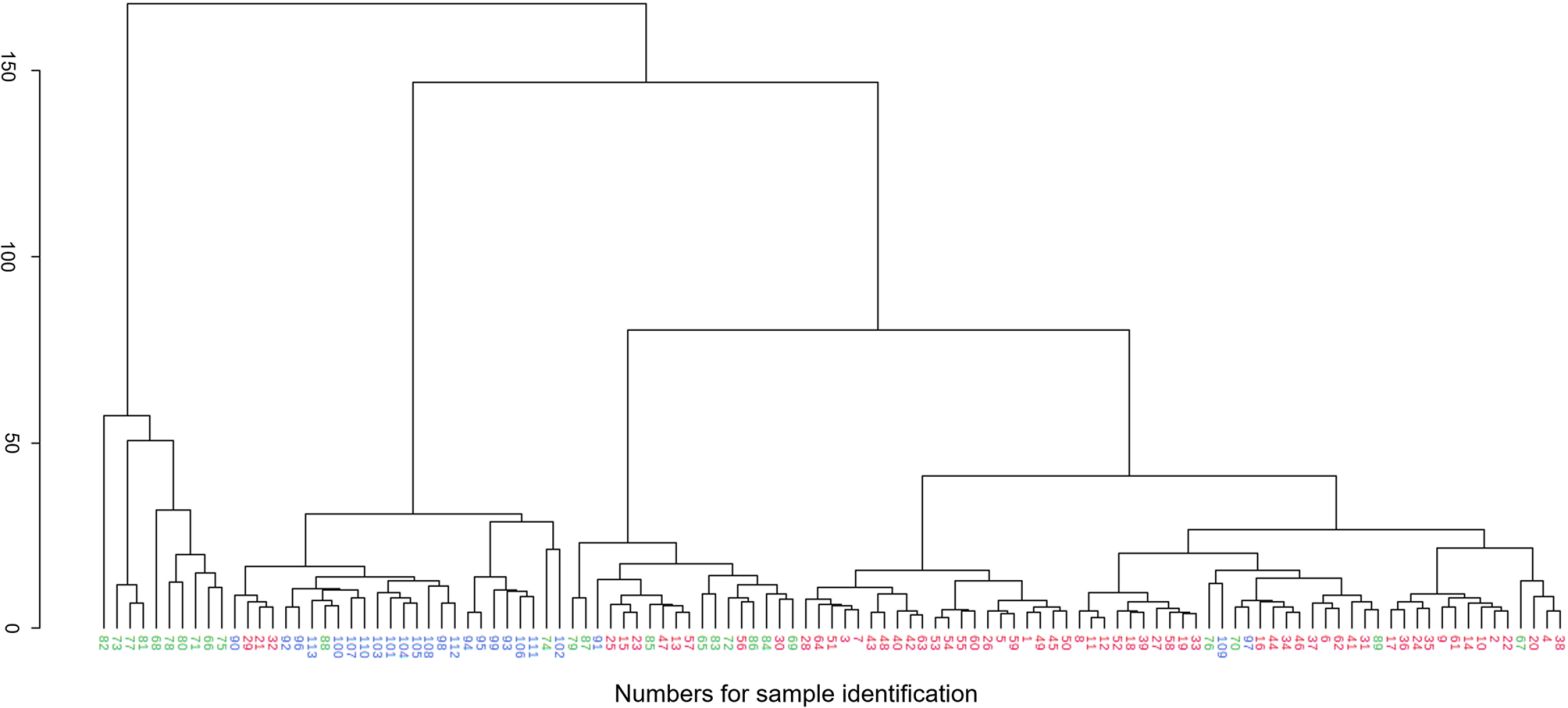
Dendrogram of hierarchical cluster analysis of serum metabolomic results from dogs with cPSS (blue, n = 24), HLEA (green, n = 25), and the combined Control group (red, n = 64). Each number on the x-axis reflects a serum sample. The y-axis shows the similarity levels expressed as Euclidean distances. Horizontal and vertical lines depict clustering of samples and differences of the distances, respectively.

Hierarchical cluster analysis using the Ward clustering algorithm and the Euclidean distance measure was used to classify samples into homogenous clusters based on similarities of their serum metabolomic results (Fig. 6). Most of the samples from dogs with cPSS and Controls could be assigned to two distinct clusters, indicating within-group similarities. In contrast, only 40% of HLEA samples formed a separate cluster, and many HLEA samples assigned to clusters of control or cPSS samples.

Several machine learning methods were applied to predict the correct assignment of samples based solely on their serum metabolomics data and without considering any routine clinical pathology results. Depending on the model used, a correct classification was achieved in 83–94% of samples using 10-fold cross-validation (Supplementary Table S3). For instance, the simple logistic regression model^40^ was able to classify 106 of 113 samples correctly based solely on the serum metabolomic analysis (Table 1).

**Table 1:** Simple logistic regression model to classify dogs with hepatopathies and dogs in the combined control group compared to the clinicopathologically assigned groups. 93.8% samples were correctly assigned using 10-fold cross-validation and solely metabolomics data.

Clinicopathologically assigned groups	Dogs, n	Groups assigned by machine learning		
		cPSS	HLEA	Controls
cPSS	24	23	0	1
HLEA	25	2	21	2
Controls	64	0	2	62

## Discussion

While measurements of singular metabolic parameters have been previously used to clinically assess patients in human and veterinary medicine, more recent comprehensive analytic test methods are now available to assess the metabolome in health and disease^19,20^. For example, while amino acid analyzers or gas chromatography can be used individually or in combination to identify and quantify distinct groups of low-molecular weight metabolites, ^1^H NMR spectroscopy can assess a broad range of metabolites without the need for tedious sample preparation steps like derivatization and only requires a very small sample volume (≤ 100µL). In this study, we have applied this novel metabolomics platform to identify metabolic abnormalities in serum samples from dogs with either cPSS (n = 24) or HLEA (n = 25) for comparison with dogs with blood test results within age-dependent reference intervals (n = 64). Hierarchical cluster analysis indicates within-group similarities in serum metabolomic results for samples from both the cPSS and Control groups. However, samples from dogs with HLEA were more randomly distributed within the cPSS and the Control group clusters, showing that the serum metabolomes of dogs with HLEA are more heterogeneous (Fig. 6). Notably, the broader clusters of HLEA observed by PCA (Fig. 2b) and PLS-DA (Fig. 3a) likely reflect the variety of hepatic diseases that may be included in this group (no histopathological results available). Moreover, dogs with cPSS have hepatic atrophy, while the liver of dogs with HLEA may show inflammation, destruction, and regeneration^12^.

The ^1^H NMR spectroscopy platform used here quantifies 105 metabolites and calculates 18 fractions and ratios of amino acids and/or fatty acids in canine serum samples. For the younger and older adult Control groups, the medians and the 25th and 75th percentiles of serum metabolomic parameters fell largely into the recently published normal serum reference intervals for dogs of all ages^30^. The minor differences may relate to the smaller number of samples and different sample handling practices and demographics compared to the validation study^30^. The serum metabolomic results for both Control groups (≤ 3 yr versus > 3 yr) showed only minor differences and near-complete overlapping of clusters in PCA and thus were combined in further analyses (Fig. 2a).

Most serum metabolite concentrations measured by ^1^H NMR spectroscopy showed differences between the three groups, and distinct metabolomic patterns differentiated the two hepatic conditions from each other, as well as from controls. Among the 123 metabolomic parameters measured, 100 were significantly different in dogs with cPSS, HLEA, and/or the combined Control group. Overall, many abnormalities appeared marginal compared to the established large reference intervals^30^. Interestingly, most lipid-related metabolic abnormalities moved in the opposite direction for cPSS and HLEA. Other parameters, for instance, phenylalanine concentrations, moved in varying degrees in the same direction for both hepatopathies. Finally, as these canine hepatopathies reflect similarities to human conditions, our findings may also be insightful for differentiating hepatopathies in humans. Applying pattern recognition methods and successfully classifying distinct diseases solely by metabolites could be an alternative to otherwise invasive diagnostic approaches, especially with hepatopathies.

The metabolomic changes observed in this study measured by ^1^H NMR spectroscopy in canine hepatopathies confirm and expand prior metabolomic studies, which were carried out using smaller sample sizes and primarily focused on other specific groups of metabolites^25,26^. One study identified 50 metabolites with significant differences, however, no metabolite concentrations and only measured peak heights were made available^25^. As age-matched control samples were missing in prior investigations^25,26^, our findings that there are no major differences in metabolites or metabolic patterns in a comparative study of younger and older adult control dogs will aid interpretation of prior and future studies.

Machine learning models, solely utilizing the serum metabolomic parameters, correctly assigned a major proportion of samples to the groups of control dogs and dogs with either cPSS or HLEA (Table 1, Supplementary Table S3). In human patients with non-alcoholic fatty liver disease, non-alcoholic steatohepatitis, or non-alcoholic steatohepatitis associated cirrhosis, machine learning models were applied to differentiate these diseases and showed good accuracy^49^. In the future, these methods could be further explored for diagnosis and monitoring of patients with various hepatopathies. As we focused on dogs with hepatopathies in this study, we did not include the assessment of serum samples from dogs with other disorders. Such analyses will be needed to better categorize disease states by multivariate analyses, including machine learning. In addition, the broad clusters of the HLEA group in PCA and PLS-DA indicate that the study of additional specific hepatopathies and severity states are required to determine if metabolomic cluster analyses and machine learning can differentiate them with or without routine diagnostic analysis. Due to the use of left-over samples, the information on clinical features, diet, and fasting/feeding status of the dogs was limited. Therefore, further prospective studies with clinically and histopathologically well defined groups, preferably with follow-up during and after treatment, are needed to confirm our findings.

Below, selective changes in specific metabolite groups in dogs with cPSS and HLEA are discussed:

### Amino acid abnormalities

In contrast to dedicated amino acid high-performance liquid chromatography (HPLC) analyzers which require more sample processing, the ^1^H NMR spectroscopy platform used here measures only nine amino acids. Still, those measured (e.g., BCAAs, phenylalanine, and tyrosine) are of particular interest when assessing hepatopathies. However, other metabolomic methods able to quantify more serum amino acids revealed that alterations in cysteine, serine, methionine, and threonine were additional distinguishing parameters of cPSS and hepatitis in small groups of dogs^25^. As serum serine and methionine concentrations changed during medical treatment in dogs with cPSS^27^, they could also be considered for further studies in hepatopathies. Indeed, the BCAAs (leucine, isoleucine, and valine) and AAAs (phenylalanine and tyrosine) concentrations were altered in both hepatopathy groups of this study. Both groups showed high serum concentrations of phenylalanine, and dogs with cPSS also showed high concentrations of tyrosine (Fig. 4b and 4c) and histidine. In contrast, concentrations of total BCAAs were low in dogs with cPSS, while dogs with HLEA had high concentrations of total BCAAs compared to the Control group (Fig. 4a). Similar changes in the amino acid panel of dogs with cPSS have previously been reported using other methods^25,27,50,51^. While, increased phenylalanine concentrations have been previously found in dogs with either hepatitis or liver neoplasia, the degree of elevation could not differentiate the two conditions^52^. Likewise, in our study, the difference between serum phenylalanine concentrations in dogs with cPSS and HLEA did not reach significance by univariate analysis (Fig. 4b), but phenylalanine was still a relevant marker in PLS-DA to distinguish between the two hepatopathies.

Accordingly, the BCAA to AAA ratio was reduced, which also occurs in humans with liver cirrhosis^53–57^. The increases of tyrosine and phenylalanine in our canine study may reflect impaired hepatic metabolism and shunting of blood, as both are mainly catabolized in the liver^54,58^. The reduction in BCAA concentrations with hepatopathies may be due to several factors, including malnutrition, portosystemic shunting, increased gluconeogenesis, and conversion of ammonia into glutamine in the skeletal muscle^56,57^. The altered BCAA and AAA concentrations may contribute to hepatic encephalopathy^59–61^ and to decreased albumin synthesis^62^. These amino acid changes may be guiding the supplementation and restriction of amino acids in canine hepatopathies. A reduction in phenylalanine and tyrosine, and a rise in valine concentrations were achieved in dogs after shunt surgery, indicating the potential for metabolic reversibility^27^.

### Abnormalities of glycolysis related metabolites

The increased serum lactate concentrations in dogs with cPSS and HLEA observed in this study were marginal and values stayed mostly in the established reference intervals^30^. Humans with liver cirrhosis^63^ or fulminant liver failure show elevated lactate concentrations^64,65^, potentially related to impaired hepatic lactate clearance with reduction of hepatic gluconeogenesis and increased glycolysis^66,67^. In people with non-alcoholic fatty liver disease, increased plasma levels of citrate, an intermediary metabolic substrate, were observed and associated with increased breakdown of fatty acids^68^. Also, human patients with fibrosis due to chronic hepatitis C^69^ and liver cirrhosis^70^ showed increased citrate concentrations. Interestingly, dogs with cPSS had slightly diminished citrate concentrations, which deserves further examination in the future. Serum acetate concentrations were increased in both hepatopathies, while only dogs in the HLEA group showed increased fatty acid concentrations. The liver hardly metabolizes any free acetate, which is instead generated during accelerated ketogenesis from fatty acid oxidation^71^ and higher levels could be associated with a ketogenic situation. However, glucose and glycolytic parameters, e.g., lactate, pyruvate, and their ratio may be affected by sample handling and storage as they are sensitive to post collection metabolic activity and thus values may not properly reflect the in vivo situation^72^.

Finally, in a small study of serum from dogs with cPSS and chronic hepatitis, an alternative metabolomics platform (gas chromatography-MS) suggested that a slight increase in the serum xylitol peak height might be one of the main metabolic determinants in differentiating hepatopathies from healthy controls^25^. However, in a human metabolomics study, decreased xylitol serum concentrations were found in patients with hepatitis B^73^. Xylitol, which was not measured in this ^1^H NMR study, is an intermediate product of uronic acid pathway and is needed for the production of xylulose. In contrast to plants, concentrations of xylitol in mammals seem to be low due to faster metabolism^74^. The biological importance of xylitol in liver disease remains unknown.

### Lipid metabolism abnormalities

In contrast to other metabolomic analyzers, the ^1^H NMR platform can quantify a larger number of lipids, lipoproteins, and fatty acids. However, as ^1^H NMR spectroscopy has not been utilized extensively to date in dogs, little has been published on the impact of various canine hepatopathies on these serum metabolite concentrations. It has been previously shown that cholesterol concentrations can be low in cPSS and other hepatopathies with impaired liver function^10^, as was also observed in the cPSS group of our study, albeit values were still in the reference interval. In contrast, many diseases, including chronic hepatitis^75^, cholangitis/cholangiohepatitis^76^, and cholelithiasis^77^, which are associated with elevated serum liver enzyme activities, can show hypercholesterinemia and hypertriglyceridemia. Although the specific causes of hepatic disease of dogs in the HLEA group were not determined in this study (no liver histopathology), dogs within this group had high serum cholesterol concentrations. Hypercholesterinemia in liver disease in humans and animals is related to cholestasis^10,13^, as well as to increased hepatic synthesis and decreased lecithin-cholesterol acyltransferase^13^. Moreover, while concentrations of most lipoproteins were also elevated in dogs with HLEA, they remained normal or low in dogs with cPSS compared to the Control group. However, concentrations of small-HDL particles, as well as cholesterol, lipids, and phospholipids within these particles, were lower in dogs with HLEA and even lower in dogs with cPSS compared to the Control group. Being the smallest lipoproteins, HDL are secreted by the liver and intestine and transport cholesterol back to the liver^78^. Low HDL cholesterol levels are seen in human patients with liver diseases such as acute viral hepatitis and severe cholestasis^79^, as well as liver cirrhosis, and can arise from multiple causes^80–82^. Lower HDL cholesterol concentrations were also associated with decreased survival time in patients with liver cirrhosis and acute gastrointestinal bleeding^81^ and also predicted the development of complications associated with liver cirrhosis^82^. Indeed, lower HDL concentrations were suggestive of more severe chronic liver failure in human patients^83^. As small-HDL and large-HDL particles, and some of their associated lipids and cholesterols had discriminative power in our PLS-DA (Fig. 3b), further studies are warranted to evaluate not only the discriminatory power between groups but also to study the association of those parameters with severity and recovery or progression of canine hepatopathies.

It is advantageous to assess individual fatty acids by both absolute concentrations and their concentrations relative to total fatty acid concentrations to better reflect changes of individual fatty acids^32^. While the absolute concentrations of individual free and esterified fatty acids uniquely determined by the ^1^H NMR platform here reflect the changes in total concentrations of triglycerides and cholesterol, the changes in relative concentrations of individual fatty acids vary. Total fatty acid concentrations and nearly all individual fatty acid concentrations (except absolute concentrations of arachidonic, docosapentaenoic, and docosahexaenoic acid) were elevated in dogs with HLEA compared to the Control group. In contrast, dogs with cPSS showed reduced concentrations of total and all individual fatty acids (except docosahexaenoic acid) compared to the Control group. In a study of hepatectomized dogs, relative serum concentrations of arachidonic acid were reduced and associated with impaired arachidonic acid synthesis^84^. Likewise, humans with various liver diseases have reduced arachidonic and polyunsaturated fatty acids, such as linoleic or linolenic acid, likely due to impaired liver metabolism and shunting^79,85,86^. While there was no observed difference in relative concentrations of linoleic acid in any group in the present study, dogs with cPSS and HLEA showed lower relative concentrations of arachidonic acid, which was most apparent in dogs with cPSS (Fig. 4e).

### Inflammatory biomarker

Glycoprotein acetyls represent a heterogenous signal of N-acetylglucosamine residues within certain acute-phase proteins, mainly α_1_-acid glycoprotein, α_1_-antitrypsin, α_1_-antichymotrypsin, haptoglobin, and transferrin^87,88^. Indeed, measurement of GlycA by ^1^H NMR spectroscopy serves as an inflammatory biomarker^87–90^, and in humans serum concentrations of GlycA correlate with increases in other inflammatory biomarkers, such as high-sensitivity C-reactive protein, fibrinogen, interleukin-6, and serum amyloid A concentrations^87,89–91^. Several diseases have been associated with increased GlycA concentrations, including type 2 diabetes, cardiovascular diseases, autoimmune diseases, obesity, and others^92–96^.

Normal ranges of serum GlycA concentrations have been assessed by ^1^H NMR spectroscopy in dogs^30^ and were increased in phenobarbital-treated dogs^97^. In addition, serum α_1_-acid glycoprotein concentrations were elevated in dogs with acute hepatopathies^98^. Furthermore, in one study^99^ serum haptoglobin concentrations were found to be high and low in dogs with hepatitides and end-stage liver disease, respectively. However, in another study serum haptoglobin concentrations remained unchanged in dogs with hepatitis or cPSS^100^. While no acute-phase protein concentrations were measured in our study, the serum GlycA concentrations were normal and high in dogs with cPSS and HLEA, respectively (Fig. 4f). This is consistent with the different disease etiology as cPSS arises from a non-inflammatory vascular anomaly, while the HLEA group likely included dogs with inflammatory hepatopathies.

### Limitations

As mentioned throughout the manuscript, this study has several limitations based on its use of left-over samples submitted to a veterinary reference laboratory. Dogs in the two hepatopathy groups as well as control dogs were carefully selected based upon routine blood test results and other information gathered from submission forms. For the cPSS group, clinical information gathered from the attending veterinarians was also assessed. However, the animals were not directly examined by the authors and histopathological diagnoses were not available to further define the origin and underlying etiology of the elevated serum liver enzyme activities in the HLEA group. Serum bile acid concentrations were not available for dogs with HLEA, as this parameter would be confounded by hyperbilirubinemia in many cases. Dogs with HLEA could also have acquired portosystemic shunts. While dogs in the Control group showed routine clinical chemistry and hematological test results within the reference intervals, occult diseases or diseases unable to be detected via routine blood test results cannot be fully excluded. However, routine blood and metabolomic test results of the control dogs were tightly grouped and well within the published normal reference intervals.

While guidelines and general veterinary practice recommend collection of blood samples from fasted dogs for diagnostic testing, the fasting status of the dogs could not be definitively determined. Typically dogs with cPSS are inappetent^101^ and thus likely reflect fasting samples. Furthermore, postprandial bile acid results are not needed when high fasting serum bile acid concentrations were found on first sample for the diagnosis of cPSS. Finally, we only included samples with high bile acid concentrations > 40 µmol/L, which would be indicative of cPSS in both fasting and postprandial samples.

Samples are typically sent overnight to reference laboratories from veterinary practices (laboratory’s courier service). The stability of metabolites in canine serum samples was demonstrated in the validation study of the ^1^H NMR method^30^. However, some metabolite concentrations can change within minutes to hours of collection^72^ and different travel times might have caused some variation in metabolite values.

Despite these limitations, the two hepatopathy and Control groups clearly differed by routine as well as metabolomic parameters. As major differences were noted, further studies using more standardized sample acquisition from fasted dogs with more expedient serum separation and freezing are warranted. In the future, this technique may allow further differentiation of these groups into subgroups based upon liver histopathology or into specific types of cPSS.

## Conclusion

Targeted metabolomic ^1^H NMR analysis revealed major metabolomic abnormalities in the serum of dogs with high liver enzyme activities and dogs with congenital portosystemic shunts. Among altered metabolites were amino acids, such as BCAAs, phenylalanine and tyrosine, but also fatty acids, lipids, GlycA, and others. Identification of altered biomarkers and metabolic pathways may improve our understanding of the pathophysiology and be helpful for diagnostics of hepatopathies in dogs. Potentially, further studies evaluating the effect of treatment on serum metabolomics might identify additional parameters to be used as biomarkers in therapy management and prognosis. Serum metabolomic patterns of both canine hepatopathy groups varied markedly and machine learning models showed great potential for classifying hepatopathies based solely on the metabolomics data. While our study used left-over samples, additional studies with distinct hepatopathies beyond cPSS and histopathological diagnoses are warranted to confirm and expand our promising findings and determine if singular parameters such as phenylalanine or tyrosine might be used in routine diagnostics to support laboratory differentiation between liver diseases, thereby advancing precision medicine.

## Supplementary Information

Supplementary Information.

## Data Availability

The datasets generated and analyzed during this study are available from the corresponding author upon reasonable request.
