# Supplementary material for: Metabolomic serum abnormalities in dogs with hepatopathies
Source: Sci Rep. 2022 Mar 29;12:5329. doi: 10.1038/s41598-022-09056-5 (PMC8964695; doi:10.1038/s41598-022-09056-5)
Supplement: Supplementary file 1 — Supplementary Information. [file 41598_2022_9056_MOESM1_ESM.docx]

**Metabolomic serum abnormalities in dogs with hepatopathies**

**Carolin A. Imbery**^1,2^**, Frank Dieterle**^3^**, Claudia Ottka**^4,5^**, Corinna Weber**^2^**, Götz Schlotterbeck**^3^**, Elisabeth Müller**^2^**, Hannes Lohi**^4,5^**, Urs Giger**^1,6,*^

^1^Vetsuisse Faculty, University of Zürich, Zürich, 8057, Switzerland

^2^Laboklin GmbH & Co. KG, Bad Kissingen, 97688, Germany

^3^Institute for Chemistry and Bioanalytics, School of Life Sciences, University of Applied Sciences Northwestern Switzerland, Muttenz, 4132, Switzerland

^4^PetMeta Labs Oy, Helsinki, 00300, Finland

^5^University of Helsinki and Folkhälsan Research Center, Helsinki, 00250, Finland

^6^Section of Medical Genetics, University of Pennsylvania, Philadelphia, PA 19104, USA

|  | | **cPSS** | **HLEA** | **Controls ≤3 yr** | **Controls >3 yr** | ***p*-value** |
| --- | --- | --- | --- | --- | --- | --- |
| **Number of dogs** | | 24 | 25 | 32 | 32 |  |
| **Age**, years, median (range) | | 1.0 (0.3–3.0)^a^ | 9.5 (2.5–15.0)^b^ | 0.9 (0.5–3.0)^a^ | 6.0 (3.3–11.0)^b^ | *<.*001 |
| **Breeds** | |  |  |  |  |  |
| Mixed breed, n | | 4 | 10 | 11 | 8 |  |
| Others (≤3 dogs/breed), n | | 20 | 14^‡^ | 21 | 24 |  |
| **Sex** | |  |  |  |  |  |
| Males, intact/castrated, n | | 10/2 | 7/9 | 12/3 | 11/8 |  |
| Females, intact/spayed, n | | 11/1 | 2/6^‡^ | 13/4 | 7/5^‡^ |  |
| **Serum parameters**, median (range) | Reference interval |  | | | |  |
| ALT | 0–55 U/L | 67.0 (37.7–258.9)^a^ | 612.6 (450.4–1883.9)^b^ | 17.2 (6.5–29.1)^c^ | 22.7 (11.4–50.8)^c^ | <.001 |
| AST | 0–25 U/L | 24.7 (13.7–245.2)^a^ | 94.9 (11.1–517.7)^a^ | 11.7 (7.2–17.6)^b^ | 9.5 (6.0–15.6)^b^ | <.001 |
| ALP | 0–108 U/L | 71.5 (12.0–560.0)^a^ | 281.0 (29.0–865.0)^a^ | 35.0 (9.0–215.0)^b🞵^ | 16.0 (7.0–45.0)^c^ | <.001 |
| GLDH | 0–6 U/L | 4.2 (0.1–54.8)^a^ | 115.1 (15.3–301.5)^b^ | 1.7 (0.1–3.7)^c^ | 1.9 (0.5–5.8)^c^ | <.001 |
| Bilirubin | 0–3.4 µmol/L | 2.1 (0.1–4.3)^ab^ | 2.6 (0.6–48.0)^a^ | 1.2 (0.1–3.4)^b^ | 1.3 (0.1–2.7)^b^ | <.001 |
| Bile acid | 0–15 µmol/L | 129.9 (42.5–332.5)^a^ | ND | 0.7 (0.1–7.1)^b✝^ | ND | <.001 |

**Supplementary Table S1** Demographic data of 113 serum samples from dogs with cPSS (n=24), HLEA (n=25), and both Control groups (n=32, each).

^‡^Breed or sex were not reported for one dog.

^🞵^Two dogs, both 5 months old, showed elevated ALP activity (125 U/L and 215 U/L).

^✝^Bile acid concentrations determined in 24 of 32 dogs in Control ≤3 yr group.

Reference intervals used were established at Laboklin.

Groups were compared by Mann-Whitney U test for bile acid concentrations, by Kruskal-Wallis test for other serum parameters and age, and by chi-square test for sex and neutering status. Level of significance was set at *p*<.05. Results with different letter superscripts (^a, b, c^) in the same line are significantly different from each other. The *p*-values shown in the final column refer to Kruskal-Wallis or Mann-Whitney U test.

The true effect of breed could not be calculated, due to the low number of dogs per breed and group, but there were no obvious differences.

There was no statistical difference in sex. A significant difference in neutering status between the groups was observed (*p*<.001), post hoc comparisons found a higher number of neutered animals in HLEA.

ALT, alanine transaminase; ALP, alkaline phosphatase; AST, aspartate transaminase; cPSS, congenital portosystemic shunt; HLEA, high liver enzyme activities; GLDH, glutamate dehydrogenase; ND, not determined; yr, years.

| **Parameter** | **Reference interval**^†^ | **cPSS** | **HLEA** | **Control combined** | ***p-*value**^‡^ | **Controls ≤3 yr**^🞵^ | **Controls >3 yr**^🞵^ |
| --- | --- | --- | --- | --- | --- | --- | --- |
| **Amino acids**, median (range) | |  |  |  |  |  |  |
| Phenylalanine | 0.03-0.07 mmol/L | 0.12 (0.04-0.17)^a^ | 0.07 (0.04-0.11)^a^ | 0.04 (0.03-0.06)^b^ | <.001 | 0.05 (0.03-0.06) | 0.04 (0.03-0.06) |
| Tyrosine | 0.04-0.09 mmol/L | 0.11 (0.06-0.15)^a^ | 0.07 (0.04-0.11)^b^ | 0.06 (0.04-0.10)^b^ | <.001 | 0.06 (0.04-0.10) | 0.06 (0.04-0.09) |
| Histidine | 0.05-0.10 mmol/L | 0.10 (0.07-0.14)^a^ | 0.06 (0.04-0.12)^b^ | 0.07 (0.04-0.12)^b^ | <.001 | 0.07 (0.06-0.12) | 0.07 (0.04-0.09) |
| Glutamine | 0.64-1.02 mmol/L | 0.81 (0.54-1.19) | 0.63 (0.35-1.00) | 0.65 (0.46-0.97) | >.05 | 0.62 (0.46-0.82) | 0.68 (0.53-0.97) |
| Total BCAA | 0.24-0.52 mmol/L | 0.24 (0.14-0.77)^a^ | 0.48 (0.15-0.69)^b^ | 0.35 (0.21-0.54)^c^ | <.001 | 0.33 (0.21-0.48) | 0.38 (0.25-0.54) |
| Leucine | 0.08-0.19 mmol/L | 0.07 (0.03-0.25)^a^ | 0.14 (0.06-0.21)^b^ | 0.12 (0.07-0.18)^b^ | <.001 | 0.10 (0.07-0.17) | 0.12 (0.07-0.18) |
| Isoleucine | 0.04-0.09 mmol/L | 0.05 (0.03-0.14)^a^ | 0.08 (0.03-0.15)^b^ | 0.07 (0.04-0.10)^a^ | <.05 | 0.06 (0.04-0.09) | 0.07 (0.05-0.10) |
| Valine | 0.11-0.25 mmol/L | 0.11 (0.06-0.38)^a^ | 0.24 (0.07-0.38)^b^ | 0.17 (0.11-0.26)^c^ | <.001 | 0.16 (0.11-0.25) | 0.19 (0.12-0.26) |
| Alanine | 0.22-0.60 mmol/L | 0.24 (0.14-0.39) | 0.30 (0.10-0.55) | 0.28 (0.18-0.47) | >.05 | 0.29 (0.18-0.47) | 0.27 (0.19-0.44) |
| Glycine | 0.13-0.45 mmol/L | 0.38 (0.07-0.59) | 0.37 (0.05-0.71) | 0.41 (0.11-0.76) | >.05 | 0.42 (0.28-0.76) | 0.40 (0.11-0.62) |
| Alanine/BCAA | 0.6-1.6 | 0.9 (0.5-1.7)^a^ | 0.7 (0.4-1.4)^b^ | 0.8 (0.5-1.4)^a^ | <.05 | 0.9 (0.6-1.4) | 0.7 (0.5-1.2) |
| Alanine/Valine | 1.2-3.5 | 2.0 (1.0-3.5)^a^ | 1.3 (0.8-2.8)^b^ | 1.6 (1.0-2.8)^c^ | <.001 | 1.8 (1.1-2.8) | 1.5 (1.0-2.8) |
| BCAA/Tyrosine | 3.8-9.2 | 2.2 (1.1-6.3)^a^ | 6.0 (1.7-12.1)^b^ | 5.5 (3.8-8.7)^b^ | <.001 | 5.1 (3.8-8.7) | 5.8 (3.8-8.6) |
| Phenylalanine/Tyrosine | 0.5-1.0 | 1.0 (0.7-1.6)^a^ | 1.0 (0.6-1.7)^a^ | 0.7 (0.5-1.1)^b^ | <.001 | 0.7 (0.5-1.1) | 0.7 (0.5-1.0) |
| Glycine/Valine | 0.7-3.0 | 3.3 (0.7-6.7)^a^ | 1.6 (0.1-6.1)^b^ | 2.3 (0.8-4.8)^b^ | <.05 | 2.5 (1.5-4.8) | 2.1 (0.8-3.7) |
| Glycine/BCAA | 0.3-1.5 | 1.4 (0.3-2.9) | 0.8 (0.1-2.7) | 1.2 (0.3-2.4) | >.05 | 1.2 (0.8-2.4) | 1.0 (0.3-1.8) |
| **Fatty acids**, median (range) | |  |  |  |  |  |  |
| Total fatty acids | 9.7-21.0 mmol/L | 11.7 (7.2-18.1)^a^ | 21.4 (10.4-35.0)^b^ | 15.0 (10.2-20.3)^c^ | <.001 | 15.0 (12.6-19.8) | 15.0 (10.2-20.3) |
| Polyunsaturated fatty acids | 4.7-11.1 mmol/L | 5.3 (3.2-9.3)^a^ | 10.8 (4.9-18.6)^b^ | 7.7 (5.0-11.1)^c^ | <.001 | 8.0 (6.1-10.5) | 7.6 (5.0-11.1) |
| Omega-6 fatty acids | 4.1-9.8 mmol/L | 4.9 (2.8-8.6)^a^ | 10.1 (4.3-17.7)^b^ | 7.1 (4.5-9.7)^c^ | <.001 | 7.2 (5.3-9.5) | 6.9 (4.5-9.7) |
| Arachidonic acid | 1.4-3.6 mmol/L | 1.3 (0.3-2.7)^a^ | 3.3 (1.0-6.9)^b^ | 2.5 (1.5-4.0)^b^ | <.001 | 2.8 (1.5-3.6) | 2.4 (1.6-4.0) |
| Linoleic acid | 2.5-5.9 mmol/L | 3.4 (1.3-5.4)^a^ | 6.0 (2.6-10.2)^b^ | 4.2 (2.7-5.8)^c^ | <.001 | 4.2 (3.5-5.7) | 4.2 (2.7-5.8) |
| Omega-3 fatty acids | 0.4-1.6 mmol/L | 0.5 (0.2-1.0)^a^ | 0.9 (0.2-2.8)^b^ | 0.8 (0.3-1.4)^b^ | <.01 | 0.8 (0.4-1.3) | 0.7 (0.3-1.4) |
| Docosapentaenoic acid | 0.1-0.4 mmol/L | 0.1 (0.0-0.2)^a^ | 0.2 (0.0-0.6)^b^ | 0.2 (0.1-0.4)^b^ | <.001 | 0.2 (0.1-0.3) | 0.2 (0.1-0.4) |
| Docosahexaenoic acid | 0.1-0.7 mmol/L | 0.2 (0.0-0.4) | 0.3 (0.0-0.8) | 0.3 (0.0-0.6) | >.05 | 0.3 (0.1-0.5) | 0.2 (0.0-0.6) |
| Oleic acid | 1.3-2.8 mmol/L | 1.6 (0.8-2.4)^a^ | 2.7 (0.6-4.3)^b^ | 1.9 (1.3-2.9)^c^ | <.001 | 1.8 (1.3-2.6) | 2.0 (1.3-2.9) |
| Saturated fatty acids | 3.6-7.4 mmol/L | 4.6 (3.0-6.6)^a^ | 8.3 (3.9-13.0)^b^ | 5.4 (3.9-7.2)^c^ | <.001 | 5.4 (4.7-7.2) | 5.5 (3.9-7.1) |
| **Parameter** | **Reference interval**^†^ | **cPSS** | **HLEA** | **Control combined** | ***p-*value**^‡^ | **Controls ≤3 yr**^🞵^ | **Controls >3 yr**^🞵^ |
| **Fatty acids**, median (range) | |  |  |  |  |  |  |
| Stearic acid | 1.7-3.8 mmol/L | 2.0 (1.10-3.3)^a^ | 4.0 (1.9-7.2)^b^ | 2.7 (1.8-3.6)^c^ | <.001 | 2.7 (2.2-3.5) | 2.7 (1.8-3.6) |
| Palmitic acid | 1.8-3.6 mmol/L | 2.4 (1.8-3.4)^a^ | 4.1 (2.0-5.9)^b^ | 2.7 (2.0-3.6)^c^ | <.001 | 2.7 (2.3-3.6) | 2.8 (2.0-3.5) |
| Polyunsaturated fatty acids % of total fatty acids | 47.5-54.9% | 48.1 (41.6-51.3)^a^ | 51.3 (40.0-56.8)^b^ | 51.6 (47.1-55.5)^b^ | <.001 | 52.5 (47.9-55.5) | 51.2 (47.1-54.6) |
| Omega-6 fatty acids % of total fatty acids | 41.6-47.5% | 43.2 (36.9-47.4)^a^ | 45.7 (36.3-50.7)^b^ | 46.3 (41.6-49.7)^b^ | <.001 | 46.9 (41.6-49.7) | 45.5 (41.6-49.0) |
| Arachidonic acid % of total fatty acids | 13.2-20.1% | 12.3 (4.8-16.2)^a^ | 15.7 (8.8-19.8)^b^ | 17.0 (10.9-23.9)^c^ | <.001 | 17.4 (11.5-23.9) | 16.8 (10.9-20.4) |
| Linoleic acid % of total fatty acids | 24.1-28.6% | 28.7 (15.3-32.9) | 28.6 (24.0-31.2) | 27.7 (24.8-30.4) | >.05 | 27.7 (24.8-30.2) | 27.7 (25.6-30.4) |
| Omega-3 fatty acids % of total fatty acids | 3.4-10.4% | 4.5 (1.9-7.6) | 4.1 (1.1-14.2) | 5.5 (2.1-8.6) | >.05 | 5.5 (3.0-6.9) | 5.2 (2.1-8.6) |
| Docosapentaenoic acid % of total fatty acids | 1.1-2.0% | 0.9 (0.0-1.7)^a^ | 1.1 (0.0-2.2)^a^ | 1.3 (0.8-2.1)^b^ | <.001 | 1.3 (0.8-1.7) | 1.4 (0.8-2.1) |
| Docosahexaenoic acid % of total fatty acids | 0.7-4.8% | 1.7 (0.1-4.3) | 1.3 (0.0-7.0) | 1.9 (0.1-3.5) | >.05 | 2.0 (0.8-3.2) | 1.7 (0.1-3.5) |
| Saturated fatty acids % of total fatty acids | 33.8-37.9% | 38.4 (36.1-42.3)^a^ | 36.9 (33.4-41.2)^b^ | 36.3 (34.5-38.5)^c^ | <.001 | 36.2 (34.5-38.1) | 36.3 (34.6-38.5) |
| Oleic acid % of total fatty acids | 10.7-15.1% | 13.5 (9.7-22.2) | 12.2 (5.6-18.9) | 12.2 (8.9-15.7) | >.05 | 11.7 (8.9-15.0) | 12.4 (10.7-15.7) |
| Stearic acid % of total fatty acids | 17.3-19.4% | 17.6 (15.1-20.3)^a^ | 18.5 (16.9-20.7)^b^ | 18.0 (16.1-18.9)^a^ | <.01 | 18.0 (17.1-18.8) | 18.0 (16.1-18.9) |
| Palmitic acid % of total fatty acids | 15.9-19.5% | 20.8 (18.7-25.7)^a^ | 18.3 (16.5-22.7)^b^ | 18.2 (16.7-19.9)^b^ | <.001 | 18.1 (16.9-19.9) | 18.4 (16.7-19.9) |
| Omega-6/Omega-3 fatty acids | 4.2-13.4 | 9.2 (5.7-23.4) | 10.9 (3.0-44.9) | 8.6 (5.3-22.7) | >.05 | 8.4 (6.6-15.6) | 8.9 (5.3-22.7) |
| **Glycolysis related metabolites**, median (range) | |  |  |  |  |  |  |
| Glucose | 4.4-6.8 mmol/L | 5.3 (2.8-6.1) | 4.7 (3.6-6.0) | 4.9 (2.8-6.2) | >.05 | 5.1 (2.8-6.2) | 4.8 (3.5-5.7) |
| Lactate | 1.1-3.6 mmol/L | 2.3 (0.9-5.3)^a^ | 2.5 (0.6-6.5)^a^ | 1.7 (0.8-4.2)^b^ | <.01 | 1.5 (0.8-4.1) | 1.7 (0.9-4.2) |
| Pyruvate | 0.01-0.11 mmol/L | 0.06 (0.02-0.13) | 0.06 (0.02-0.14) | 0.05 (0.02-0.12) | >.05 | 0.05 (0.02-0.12) | 0.04 (0.02-0.10) |
| Acetate | 0.02-0.04 mmol/L | 0.04 (0.02-0.08)^a^ | 0.03 (0.02-0.06)^a^ | 0.02 (0.02-0.04)^b^ | <.01 | 0.02 (0.02-0.03) | 0.03 (0.02-0.04) |
| Citrate | 0.06-0.12 mmol/L | 0.05 (0.00-0.09)^a^ | 0.08 (0.02-0.19)^b^ | 0.06 (0.01-0.09)^c^ | <.05 | 0.06 (0.01-0.08)^a^ | 0.07 (0.05-0.09)^b^ |
| **Fluid balance**, median (range) | |  |  |  |  |  |  |
| Albumin | 25-32 g/L | 25 (18-30)^a^ | 29 (24-33)^b^ | 28 (23-31)^c^ | <.001 | 27 (23-30) | 28 (24-31) |
| Creatinine | 32-103 µmol/L | 30 (11-51)^a^ | 56 (26-180)^b^ | 60 (24-97)^b^ | <.001 | 58 (24-97) | 62 (25-76) |

| **Parameter** | **Reference interval**^†^ | **cPSS** | **HLEA** | **Control combined** | ***p-*value**^‡^ | **Controls ≤3 yr**^🞵^ | **Controls >3 yr**^🞵^ |
| --- | --- | --- | --- | --- | --- | --- | --- |
| **Inflammation marker**, median (range) | |  |  |  |  |  |  |
| GlycA | 0.60-1.03 mmol/L | 0.62 (0.38-1.11)^a^ | 1.12 (0.70-1.89)^b^ | 0.66 (0.52-0.99)^a^ | <.001 | 0.66 (0.52-0.86) | 0.68 (0.54-0.99) |
| **Triglycerides**, median (range) | |  |  |  |  |  |  |
| Total triglycerides | 0.19-1.00 mmol/L | 0.35 (0.18-1.16) | 0.65 (0.06-1.97) | 0.38 (0.05-0.87) | >.05 | 0.38 (0.16-0.74) | 0.38 (0.05-0.87) |
| VLDL triglycerides | 0.00-0.70 mmol/L | 0.10 (0.00-0.49)^a^ | 0.34 (0.00-1.52)^b^ | 0.12 (0.00-0.53)^a^ | <.05 | 0.08 (0.00-0.40) | 0.13 (0.00-0.53) |
| LDL triglycerides | 0.13-0.31 mmol/L | 0.20 (0.08-0.79) | 0.17 (0.00-0.39) | 0.24 (0.04-0.35) | >.05 | 0.26 (0.14-0.35) | 0.21 (0.04-0.34) |
| HDL triglycerides | 0.00-0.08 mmol/L | 0.02 (0.00-0.14)^a^ | 0.07 (0.01-0.16)^b^ | 0.03 (0.01-0.08)^a^ | <.001 | 0.02 (0.01-0.07) | 0.03 (0.01-0.08) |
| **Cholesterol**, median (range) | |  |  |  |  |  |  |
| Total cholesterol | 3.6-10.3 mmol/L | 3.9 (2.4-9.1)^a^ | 10.6 (4.4-21.0)^b^ | 6.6 (3.8-10.2)^c^ | <.001 | 7.0 (4.3-10.2) | 6.3 (3.8-10.0) |
| Esterified cholesterol | 2.9-8.1 mmol/L | 3.2 (1.9-7.1)^a^ | 8.2 (3.5-16.6)^b^ | 5.4 (3.2-8.0)^c^ | <.001 | 5.6 (3.5-8.0) | 5.1 (3.2-8.0) |
| Free cholesterol | 0.6-2.2 mmol/L | 0.8 (0.5-2.0)^a^ | 2.3 (0.8-4.4)^b^ | 1.3 (0.6-2.2)^c^ | <.001 | 1.4 (0.8-2.2) | 1.2 (0.6-2.1) |
| VLDL cholesterol | 0.0-0.3 mmol/L | 0.1 (0.1-0.3)^a^ | 0.4 (0.0-2.8)^b^ | 0.1 (0.0-0.4)^a^ | <.001 | 0.1 (0.0-0.4) | 0.1 (0.0-0.4) |
| LDL cholesterol | 0.3-2.3mmol/L | 0.7 (0.4-2.4)^a^ | 3.0 (0.6-8.3)^b^ | 1.0 (0.3-3.4)^a^ | <.001 | 1.3 (0.3-3.4) | 0.9 (0.3-2.8) |
| HDL cholesterol | 3.2-7.9 mmol/L | 3.2 (1.5-6.7)^a^ | 6.8 (3.3-15.4)^b^ | 5.5 (3.5-7.5)^b^ | <.001 | 5.7 (4.0-7.5) | 5.3 (3.5-7.4) |
| **Total lipids**, median (range) | |  |  |  |  |  |  |
| VLDL lipids | 0.1-1.2 mmol/L | 0.3 (0.1-1.0)^a^ | 0.7 (0.2-6.0)^b^ | 0.3 (0.1-1.0)^a^ | <.001 | 0.3 (0.1-0.8) | 0.3 (0.1-1.0) |
| LDL lipids | 0.7-3.7 mmol/L | 1.4 (0.9-4.1)^a^ | 4.8 (1.2-12.5)^b^ | 1.9 (0.7-5.5)^a^ | <.001 | 2.2 (0.7-5.5) | 1.8 (0.7-4.4) |
| HDL lipids | 6.9-15.1 mmol/L | 7.1 (3.6-12.8)^a^ | 13.8 (6.9-28.8)^b^ | 11.1 (7.5-14.4)^b^ | <.001 | 11.3 (8.4-14.3) | 10.6 (7.5-14.4) |
| **Particle concentrations**, median (range) | |  |  |  |  |  |  |
| VLDL particles | 0.01-0.05 µmol/L | 0.03 (0.02-0.05)^a^ | 0.07 (0.02-0.40)^b^ | 0.03 (0.01-0.07)^a^ | <.001 | 0.03 (0.02-0.07) | 0.03 (0.01-0.06) |
| LDL particles | 0.24-1.30 µmol/L | 0.50 (0.29-1.50)^a^ | 1.76 (0.40-4.35)^b^ | 0.71 (0.26-1.98)^c^ | <.001 | 0.83 (0.26-1.98) | 0.63 (0.27-1.66) |
| HDL particles | 0.03-0.06 mmol/L | 0.03 (0.01-0.04)^a^ | 0.04 (0.01-0.09)^b^ | 0.04 (0.03-0.05)^b^ | <.001 | 0.04 (0.04-0.05) | 0.04 (0.03-0.05) |
| **Average diameter of particles**, median (range) | |  |  |  |  |  |  |
| VLDL size | 35.2-43.8 nm | 35.7 (35.0-40.3)^a^ | 39.2 (35.0-42.7)^b^ | 37.8 (35.0-43.1)^b^ | <.001 | 37.8 (35.0-42.4) | 38.3 (35.0-43.1) |
| LDL size | 22.2-23.5 nm | 23.4 (22.4-24.3)^a^ | 22.6 (22.1-23.2)^b^ | 22.6 (22.2-23.5)^b^ | <.001 | 22.6 (22.2-23.2) | 22.6 (22.2-23.5) |
| HDL size | 10.1-10.7 nm | 10.6 (10.2-11.9)^a^ | 11.0 (10.5-12.0)^b^ | 10.6 (10.2-11.0)^a^ | <.001 | 10.6 (10.33-11.0) | 10.6 (10.2-11.0) |
|  |  |  |  |  |  |  |  |
|  |  |  |  |  |  |  |  |

| **Parameter** | **Reference interval**^†^ | **cPSS** | **HLEA** | **Control combined** | ***p-*value**^‡^ | **Controls ≤3 yr**^🞵^ | **Controls >3 yr**^🞵^ |
| --- | --- | --- | --- | --- | --- | --- | --- |
| **Lipoprotein subclasses**, median (range) | |  |  |  |  |  |  |
| L-HDL cholesterol | 2.3-4.4 mmol/L | 2.1 (0.9-3.0)^a^ | 3.0 (0.5-6.7)^b^ | 3.1 (2.2-3.8)^b^ | <.001 | 3.2 (2.7-3.8) | 3.1 (2.2-3.8) |
| L-HDL esterified cholesterol | 2.0-3.8 mmol/L | 1.8 (0.8-2.5)^a^ | 2.5 (0.3-5.7)^b^ | 2.7 (1.9-3.3)^b^ | <.001 | 2.7 (2.3-3.3) | 2.6 (1.9-3.2) |
| L-HDL free cholesterol | 0.3-0.6 mmol/L | 0.3 (0.1-0.5)^a^ | 0.5 (0.2-1.0)^b^ | 0.5 (0.3-0.6)^b^ | <.001 | 0.5 (0.4-0.6) | 0.4 (0.3-0.6) |
| L-HDL lipids | 5.0-8.3 mmol/L | 4.7 (2.4-6.3)^a^ | 6.0 (2.0-12.0)^b^ | 6.3 (4.7-7.5)^b^ | <.001 | 6.4 (5.6-7.5) | 6.1 (4.7-7.3) |
| L-HDL particles | 0.02-0.03 mmol/L | 0.02 (0.01-0.02)^a^ | 0.02 (0.00-0.05)^b^ | 0.02 (0.02-0.03)^c^ | <.001 | 0.03 (0.02-0.03) | 0.02 (0.02-0.03) |
| L-HDL phospholipids | 2.48-3.94 mmol/L | 2.50 (1.45-3.43)^a^ | 3.02 (1.47-5.20)^b^ | 3.10 (2.42-3.74)^b^ | <.001 | 3.11 (2.58-3.71) | 3.06 (2.42-3.74) |
| L-HDL triglycerides | 0.00-0.03 mmol/L | 0.01 (0.00-0.03)^a^ | 0.02 (0.00-0.05)^b^ | 0.01 (0.00-0.03)^c^ | <.001 | 0.01 (0.01-0.03) | 0.01 (0.00-0.02) |
| L-LDL cholesterol | 0.07-0.68 mmol/L | 0.29 (0.16-0.71)^a^ | 0.94 (0.19-4.33)^b^ | 0.34 (0.08-1.26)^a^ | <.001 | 0.36 (0.08-1.26) | 0.31 (0.12-1.03) |
| L-LDL esterified cholesterol | 0.03-0.49 mmol/L | 0.18 (0.08-0.50)^a^ | 0.70 (0.10-3.22)^b^ | 0.22 (0.02-0.91)^a^ | <.001 | 0.25 (0.02-0.91) | 0.20 (0.06-0.76) |
| L-LDL free cholesterol | 0.04-0.20 mmol/L | 0.12 (0.09-0.21)^a^ | 0.25 (0.07-1.12)^b^ | 0.11 (0.05-0.35)^a^ | <.001 | 0.12 (0.05-0.35) | 0.11 (0.06-0.27) |
| L-LDL lipids | 0.3-1.2 mmol/L | 0.7 (0.5-1.3)^a^ | 1.5 (0.5-6.3)^b^ | 0.7 (0.4-2.1)^a^ | <.001 | 0.8 (0.3-2.1) | 0.7 (0.4-1.6) |
| L-LDL particles | 0.09-0.37 µmol/L | 0.22 (0.16-0.42)^a^ | 0.49 (0.15-1.93)^b^ | 0.23 (0.11-0.66)^a^ | <.001 | 0.25 (0.11-0.66) | 0.22 (0.12-0.54) |
| L-LDL phospholipids | 0.08-0.32 mmol/L | 0.22 (0.17-0.37)^a^ | 0.45 (0.13-1.96)^b^ | 0.22 (0.10-0.63)^a^ | <.001 | 0.23 (0.10-0.63) | 0.20 (0.10-0.51) |
| L-LDL triglycerides | 0.10-0.24 mmol/L | 0.17 (0.07-0.47) | 0.12 (0.00-0.30) | 0.18 (0.01-0.28) | >.05 | 0.20 (0.09-0.26) | 0.16 (0.01-0.28) |
| L-VLDL cholesterol | 0.00-0.13 mmol/L | 0.02 (0.00-0.11)^a^ | 0.13 (0.01-1.03)^b^ | 0.04 (0.00-0.13)^a^ | <.001 | 0.03 (0.00-0.12) | 0.04 (0.00-0.13) |
| L-VLDL esterified cholesterol | 0.00-0.06 mmol/L | 0.01 (0.00-0.05)^a^ | 0.05 (0.01-0.54)^b^ | 0.02 (0.00-0.08)^a^ | <.001 | 0.01 (0.00-0.08) | 0.02 (0.00-0.08) |
| L-VLDL free cholesterol | 0.00-0.08 mmol/L | 0.01 (0.00-0.06)^a^ | 0.05 (0.00-0.50)^b^ | 0.02 (0.00-0.06)^c^ | <.001 | 0.01 (0.00-0.06) | 0.02 (0.00-0.06) |
| L-VLDL lipids | 0.0-0.6 mmol/L | 0.1 (0.0-0.4)^a^ | 0.3 (0.0-2.6)^b^ | 0.1 (0.0-0.5)^a^ | <.001 | 0.1 (0.0-0.3) | 0.1 (0.0-0.5) |
| L-VLDL particles | 0.00-0.02 µmol/L | 0.00 (0.00-0.01)^a^ | 0.01 (0.00-0.10)^b^ | 0.00 (0.00-0.01)^c^ | <.001 | 0.00 (0.00-0.01) | 0.00 (0.00-0.01) |
| L-VLDL phospholipids | 0.00-0.11 mmol/L | 0.01 (0.00-0.07)^a^ | 0.06 (0.00-0.70)^b^ | 0.02 (0.00-0.08)^c^ | <.001 | 0.02 (0.00-0.08) | 0.02 (0.00-0.08) |
| L-VLDL triglycerides | 0.01-0.42 mmol/L | 0.02 (0.00-0.22)^a^ | 0.19 (0.00-0.86)^b^ | 0.04 (0.00-0.29)^a^ | <.001 | 0.01 (0.00-0.17) | 0.04 (0.00-0.29) |
| S-HDL lipids | 1.3-2.2 mmol/L | 1.0 (0.3-1.4)^a^ | 1.3 (0.5-2.9)^b^ | 1.5 (1.0-1.9)^c^ | <.001 | 1.5 (1.2-1.8) | 1.4 (1.0-1.9) |
| S-HDL cholesterol | 0.5-1.0 mmol/L | 0.4 (0.1-0.6)^a^ | 0.5 (0.1-1.3)^b^ | 0.6 (0.4-0.8)^c^ | <.001 | 0.6 (0.5-0.8) | 0.6 (0.4-0.8) |
| S-HDL esterified cholesterol | 0.4-0.8 mmol/L | 0.3 (0.1-0.4)^a^ | 0.4 (0.0-1.0)^b^ | 0.5 (0.3-0.7)^c^ | <.001 | 0.5 (0.4-0.6) | 0.5 (0.3-0.7) |
| S-HDL free cholesterol | 0.1-0.2 mmol/L | 0.1 (0.0-0.1)^a^ | 0.1 (0.1-0.3)^b^ | 0.1 (0.1-0.2)^b^ | <.001 | 0.1 (0.1-0.2) | 0.1 (0.1-0.1) |
| S-HDL particles | 0.01-0.02 mmol/L | 0.01 (0.00-0.01)^a^ | 0.01 (0.00-0.03)^b^ | 0.01 (0.01-0.02)^b^ | <.001 | 0.01 (0.01-0.02) | 0.01 (0.01-0.02) |
| S-HDL phospholipids | 0.72-1.19 mmol/L | 0.60 (0.22-0.84)^a^ | 0.74 (0.32-1.49)^b^ | 0.86 (0.61-1.08)^c^ | <.001 | 0.88 (0.72-1.04) | 0.82 (0.61-1.08) |

| **Parameter** | **Reference interval**^†^ | **cPSS** | **HLEA** | **Control combined** | ***p-*value**^‡^ | **Controls ≤3 yr**^🞵^ | **Controls >3 yr**^🞵^ |
| --- | --- | --- | --- | --- | --- | --- | --- |
| **Lipoprotein subclasses**, median (range) | |  |  |  |  |  |  |
| S-HDL triglycerides | 0.00-0.03 mmol/L | 0.00 (0.00-0.07)^a^ | 0.03 (0.00-0.08)^b^ | 0.00 (0.00-0.04)^a^ | <.01 | 0.00 (0.00-0.03) | 0.01 (0.00-0.04) |
| S-LDL cholesterol | 0.16-1.53 mmol/L | 0.37 (0.15-1.72)^a^ | 2.08 (0.40-4.04)^b^ | 0.74 (0.16-2.11)^c^ | <.001 | 0.86 (0.18-2.11) | 0.67 (0.16-1.74) |
| S-LDL esterified cholesterol | 0.10-1.09 mmol/L | 0.26 (0.11-1.22)^a^ | 1.49 (0.28-2.95)^b^ | 0.53 (0.11-1.52)^c^ | <.001 | 0.61 (0.12-1.52) | 0.47 (0.11-1.26) |
| S-LDL free cholesterol | 0.05-0.45 mmol/L | 0.10 (0.04-0.50)^a^ | 0.59 (0.12-1.10)^b^ | 0.21 (0.06-0.59)^c^ | <.001 | 0.25 (0.06-0.59) | 0.19 (0.06-0.49) |
| S-LDL lipids | 0.3-2.4 mmol/L | 0.7 (0.3-2.8)^a^ | 3.3 (0.7-6.3)^b^ | 1.2 (0.3-3.4)^c^ | <.001 | 1.4 (0.4-3.4) | 1.1 (0.3-2.8) |
| S-LDL particles | 0.14-0.96 µmol/L | 0.26 (0.11-1.08)^a^ | 1.29 (0.25-2.49)^b^ | 0.49 (0.13-1.31)^c^ | <.001 | 0.57 (0.15-1.31) | 0.44 (0.13-1.12) |
| S-LDL phospholipids | 0.12-0.84 mmol/L | 0.24 (0.10-0.98)^a^ | 1.17 (0.26-2.26)^b^ | 0.46 (0.12-1.19)^c^ | <.001 | 0.51 (0.13-1.19) | 0.42 (0.12-1.01) |
| S-LDL triglycerides | 0.03-0.07 mmol/L | 0.04 (0.01-0.32) | 0.05 (0.00-0.12) | 0.05 (0.03-0.09) | >.05 | 0.06 (0.04-0.09) | 0.05 (0.03-0.08) |
| S-VLDL cholesterol | 0.02-0.15 mmol/L | 0.08 (0.04-0.13)^a^ | 0.17 (0.03-1.43)^b^ | 0.07 (0.01-0.23)^a^ | <.001 | 0.08 (0.02-0.23) | 0.07 (0.01-0.21) |
| S-VLDL esterified cholesterol | 0.01-0.09 mmol/L | 0.05 (0.02-0.09)^a^ | 0.10 (0.01-0.99)^b^ | 0.04 (0.00-0.16)^a^ | <.001 | 0.04 (0.00-0.16) | 0.04 (0.00-0.15) |
| S-VLDL free cholesterol | 0.01-0.06 mmol/L | 0.02 (0.01-0.04)^a^ | 0.07 (0.02-0.44)^b^ | 0.03 (0.01-0.07)^a^ | <.001 | 0.03 (0.02-0.07) | 0.03 (0.01-0.06) |
| S-VLDL lipids | 0.1-0.4 mmol/L | 0.2 (0.1-0.3)^a^ | 0.5 (0.1-2.5)^b^ | 0.2 (0.1-0.5)^a^ | <.001 | 0.2 (0.1-0.5) | 0.2 (0.1-0.4) |
| S-VLDL particles | 0.01-0.04 µmol/L | 0.03 (0.02-0.04)^a^ | 0.06 (0.02-0.29)^b^ | 0.03 (0.01-0.06)^a^ | <.001 | 0.03 (0.01-0.06) | 0.03 (0.01-0.05) |
| S-VLDL phospholipids | 0.01-0.08 mmol/L | 0.04 (0.02-0.07)^a^ | 0.12 (0.01-0.69)^b^ | 0.05 (0.02-0.11)^a^ | <.001 | 0.05 (0.03-0.11) | 0.05 (0.02-0.09) |
| S-VLDL triglycerides | 0.02-0.16 mmol/L | 0.07 (0.00-0.12) | 0.11 (0.00-0.38) | 0.07 (0.00-0.15) | >.05 | 0.05 (0.00-0.14) | 0.07 (0.00-0.15) |
| XL-HDL cholesterol | 0.2-2.8 mmol/L | 0.8 (0.3-3.3)^a^ | 3.2 (0.8-7.4)^b^ | 1.7 (0.4-3.4)^c^ | <.001 | 1.7 (0.7-3.0) | 1.7 (0.4-3.4) |
| XL-HDL esterified cholesterol | 0.2-2.1 mmol/L | 0.6 (0.2-2.5)^a^ | 2.5 (0.6-5.8)^b^ | 1.3 (0.3-2.6)^c^ | <.001 | 1.3 (0.5-2.3) | 1.3 (0.3-2.6) |
| XL-HDL free cholesterol | 0.1-0.6 mmol/L | 0.2 (0.1-0.8)^a^ | 0.7 (0.2-1.6)^b^ | 0.4 (0.1-0.8)^c^ | <.001 | 0.4 (0.1-0.7) | 0.4 (0.1-0.8) |
| XL-HDL lipids | 0.6-5.0 mmol/L | 1.8 (0.7-6.1)^a^ | 6.2 (1.8-14.0)^b^ | 3.3 (1.0-6.4)^c^ | <.001 | 3.3 (1.5-5.7) | 3.4 (1.0-6.4) |
| XL-HDL particles | 0.0-5.8 µmol/L | 2.6 (0.8-8.0)^a^ | 7.6 (2.2-18.3)^b^ | 4.6 (1.2-8.5)^c^ | <.001 | 4.5 (2.0-7.7) | 4.6 (1.2-8.5) |
| XL-HDL phospholipids | 0.33-2.22 mmol/L | 0.93 (0.39-2.75)^a^ | 2.93 (0.99-6.53)^b^ | 1.64 (0.57-2.99)^c^ | <.001 | 1.61 (0.81-2.63) | 1.67 (0.57-2.99) |
| XL-HDL triglycerides | 0.00-0.02 mmol/L | 0.01 (0.00-0.04)^a^ | 0.02 (0.00-0.05)^b^ | 0.01 (0.00-0.02)^a^ | <.001 | 0.01 (0.00-0.02) | 0.01 (0.00-0.02) |
| XL-VLDL cholesterol | 0.00-0.06 mmol/L | 0.01 (0.00-0.06) | 0.02 (0.00-0.35) | 0.00 (0.00-0.05) | >.05 | 0.00 (0.00-0.05) | 0.01 (0.00-0.05) |
| XL-VLDL esterified cholesterol | 0.00-0.03 mmol/L | 0.00 (0.00-0.03)^a^ | 0.01 (0.00-0.17)^b^ | 0.00 (0.00-0.02)^a^ | <.05 | 0.00 (0.00-0.02) | 0.00 (0.00-0.02) |
| XL-VLDL free cholesterol | 0.00-0.05 mmol/L | 0.00 (0.00-0.04) | 0.01 (0.00-0.18) | 0.00 (0.00-0.03) | >.05 | 0.00 (0.00-0.03) | 0.00 (0.00-0.03) |
| XL-VLDL lipids | 0.0-0.3 mmol/L | 0.0 (0.0-0.3) | 0.1 (0.0-0.9) | 0.0 (0.0-0.2) | >.05 | 0.0 (0.0-0.2) | 0.0 (0.0-0.2) |
| XL-VLDL particles | 0.000-0.001 µmol/L | 0.00 (0.00-0.00) | 0.00 (0.00-0.01) | 0.00 (0.00-0.00) | >.05 | 0.00 (0.00-0.00) | 0.00 (0.00-0.00) |

| **Parameter** | **Reference interval**^†^ | **cPSS** | **HLEA** | **Control combined** | ***p-*value**^‡^ | **Controls ≤3 yr**^🞵^ | **Controls >3 yr**^🞵^ |
| --- | --- | --- | --- | --- | --- | --- | --- |
| **Lipoprotein subclasses**, median (range) | |  |  |  |  |  |  |

| XL-VLDL phospholipids | 0.00-0.05 mmol/L | 0.00 (0.00-0.05) | 0.01 (0.00-0.26) | 0.00 (0.00-0.05) | >.05 | 0.00 (0.00-0.05) | 0.00 (0.00-0.04) |
| --- | --- | --- | --- | --- | --- | --- | --- |
| XL-VLDL triglycerides | 0.00-0.17 mmol/L | 0.02 (0.00-0.15) | 0.05 (0.00-0.42) | 0.00 (0.00-0.13) | >.05 | 0.00 (0.00-0.11) | 0.02 (0.00-0.13) |

**Supplementary Table S2** Metabolomic serum parameters differing between dogs with cPSS (n=24), HLEA (n=25) and the combined Control group (n=64).

^†^Reference intervals were established in canine serum for dogs of all ages^1^.

^‡^Groups (cPSS vs HLEA vs Controls combined) were compared by Kruskal-Wallis test adjusted with Bonferroni-correction for metabolomic serum parameters. The *p*-values are shown in the sixth column (cPSS vs HLEA vs Controls combined). Results with different letter superscripts (^a, b, c^) in the same line are significantly different from each other.

^🞵^Groups (cPSS vs HLEA vs Controls ≤3 yr vs Controls >3 yr) were compared by Kruskal-Wallis test adjusted with Bonferroni-correction for metabolomic serum parameters. Post-hoc comparison between Controls ≤3 yr vs Controls >3 yr revealed only a significant difference between citrate concentrations (*p*=.049).

BCAA, branched chain amino acid; cPSS, congenital portosystemic shunt; GlycA, glycoprotein acetyls; HLEA, high liver enzyme activities; L-HDL, large high-density lipoprotein; L-LDL, large low-density lipoprotein; L-VLDL, large very-low-density lipoprotein; S-HDL, small high-density lipoprotein; S-LDL, small low-density lipoprotein; S-VLDL, small very-low-density lipoprotein; XL-HDL, extra-large high-density lipoprotein; XL-VLDL, extra-large very-low-density lipoprotein.


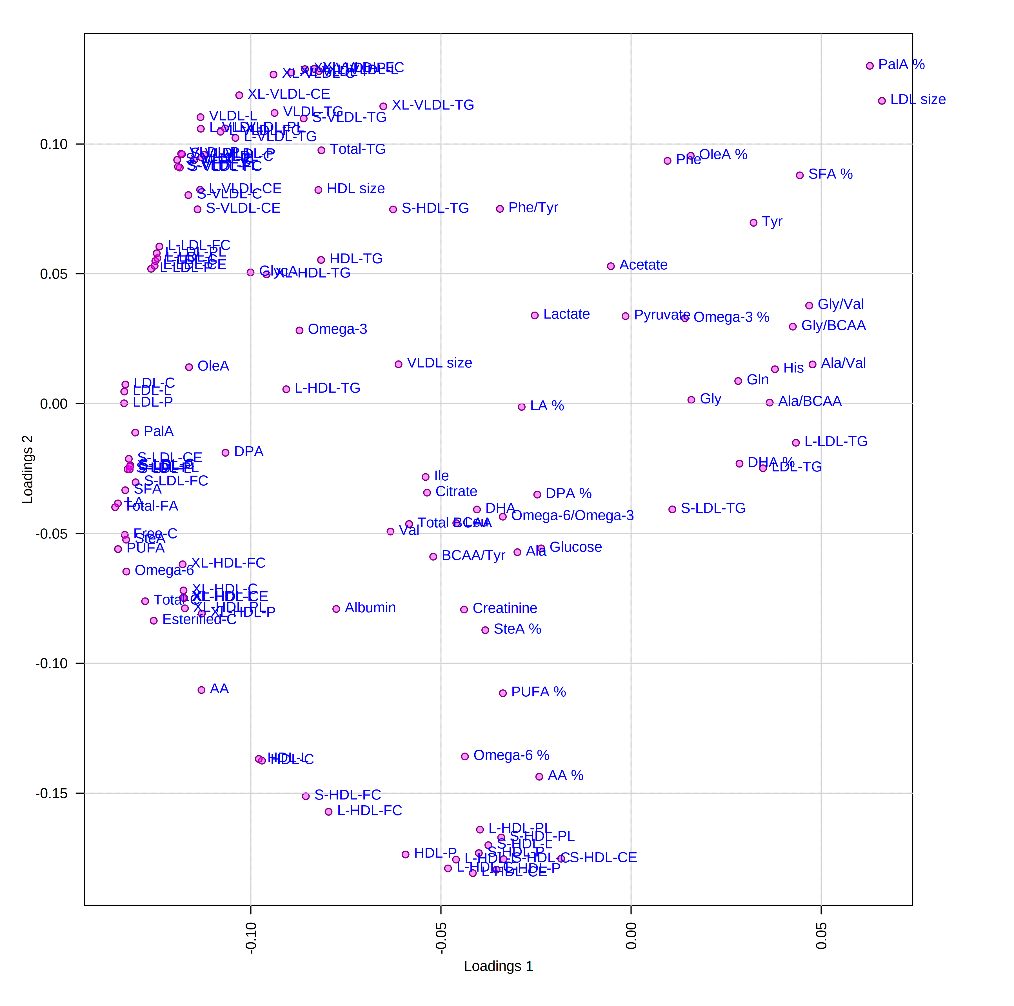
**Supplementary Figure S1** Loadings plot of principal component analysis based on metabolomic data between serum samples of dogs with cPSS (n=24) or HLEA (n=25) and combined Controls (n=64).
AA, arachidonic acid; Ala, alanine; BCAA, branched chain amino acid; C, cholesterol; CE, esterified cholesterol; cPSS, congenital portosystemic shunt; DHA, docosahexaenoic acid; DPA, docosapentaenoic acid; FC, free cholesterol; Gln, glutamine; Gly, glycine; GlycA, glycoprotein acetyls; His, histidine; HLEA, high liver enzyme activities; Ile, Isoleucine; LA, linoleic acid; Leu, leucine; L-HDL, large high-density lipoprotein; L-LDL, large low-density lipoprotein; L-VLDL, large very-low-density lipoprotein; OleA, oleic acid; P, particle; PalA, palmitic acid; Phe, phenylalanine; PL, phospholipids; PUFA, polyunsaturated fatty acids; SFA, saturated fatty acids; S-HDL, small high-density lipoprotein; S-LDL, small low-density lipoprotein; SteA, stearic acid; S-VLDL, small very-low-density lipoprotein; TG, triglycerides; Tyr, tyrosine; Val, valine; XL-HDL, extra-large high-density lipoprotein; XL-VLDL, extra-large very-low-density lipoprotein.


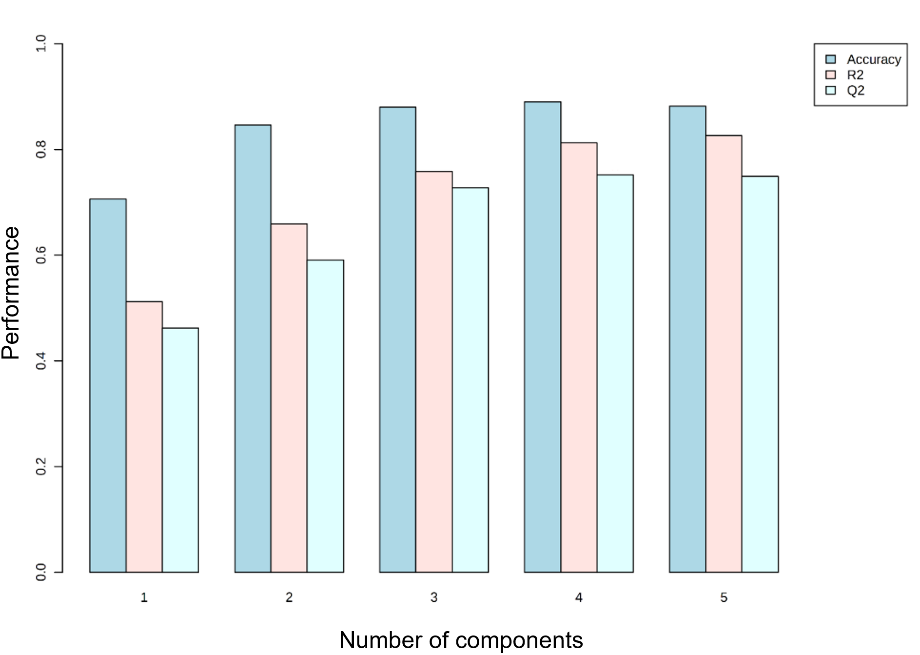
**Supplementary Figure S2** Results of the 10-fold cross-validation of the partial least squares-discriminant analysis (PLS-DA) model (Figure 3) with R^2^, Q^2^, and accuracy measures based on the number of components. Four components were chosen for the model based on the Q^2^ criterion.


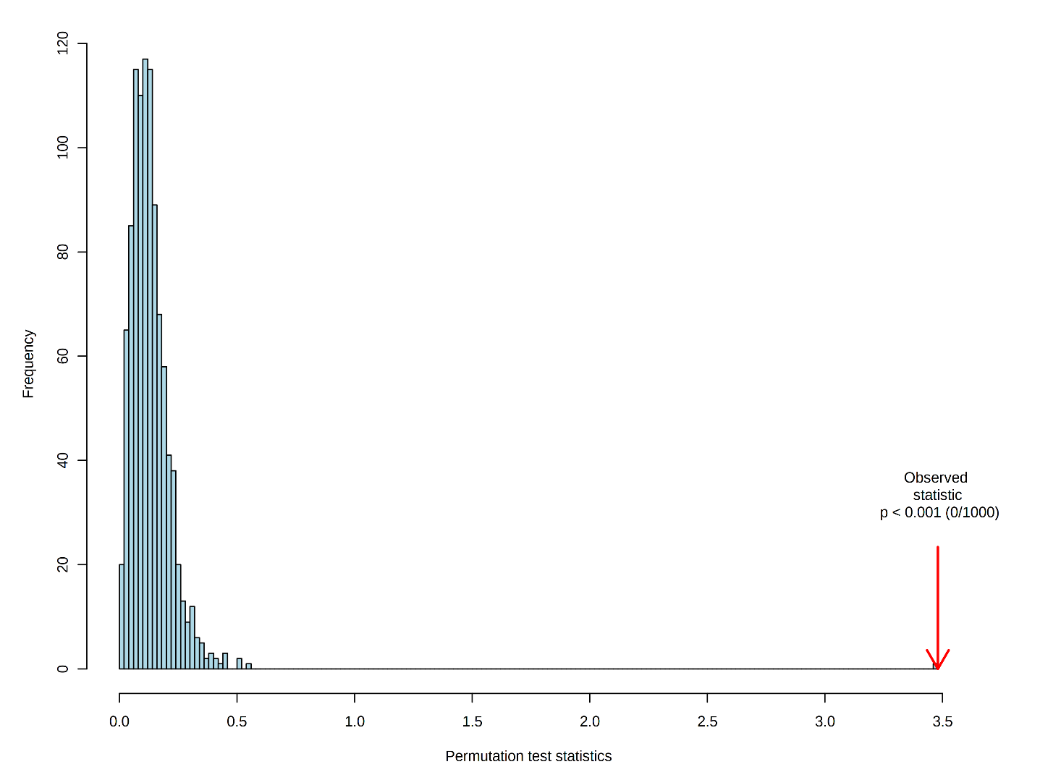
**Supplementary Figure S3** Results of a permutation test with 1000 permutations for the partial least squares-discriminant analysis (PLS-DA) model shown in Figure 3. The results that the model is not overfitting the data. None of the 1000 permutations achieved the same separation distance which is defined as the ratio of the between-group sum of the squares and the within-group sum of squares (B/W-ratio), *p*<.001.

| **Machine learning method** | **% of correct classified cases** |
| --- | --- |
| Multilayer Perceptron Classifier^2^ | 93.8 |
| Support Vector Machines - Cramer and Singer^3^ | 86.7 |
| Multinomial naïve Bayes^4^ | 86.7 |
| Simple Logistic^5^ | 93.8 |
| k-nearest neighbors algorithm^6^ | 83.2 |
| Random Forest^7^ | 86.7 |

**Supplementary Table S3** Different machine learning models classifying groups based solely on metabolomic data from serum samples of dogs with cPSS (n=24), HLEA (n=25) and the combined Control group (n=64).

cPSS, congenital portosystemic shunt; HLEA, high liver enzyme activities.

References

1. Ottka, C., Vapalahti, K., Puurunen, J., Vahtera, L. & Lohi, H. A novel canine nuclear magnetic resonance spectroscopy‐based metabolomics platform: Validation and sample handling. *Vet. Clin. Pathol.* **50,** 410–426; https://doi.org/10.1111/vcp.12954 (2021).

2. Morariu, D., Crețulescu, R. & Breazu, M. The WEKA multilayer perceptron classifier. *J. Adv. Stat. IT&C Econom. Life Sci.* **7** (2017).

3. Crammer, K. & Singer, Y. On the algorithmic implementation of multiclass kernel-based vector machines. *J. Mach. Learn. Res.* **2,** 265–292 (2002).

4. Hand, D. J. & Yu, K. Idiot's bayes: Not so stupid after all? *Int. Stat. Rev.* **69,** 385–398; https://doi.org/10.2307/1403452 (2001).

5. Sumner, M., Frank, E. & Hall, M. Speeding up logistic model tree induction. In *Knowledge Discovery in Databases: PKDD 2005. 9th European Conference on Principles and Practice of Knowledge Discovery in Databases,* edited by D. Hutchison*, et al.* (Springer Verlag, Berlin, Heidelberg, 2005), Vol. 3721, pp. 675–683.

6. Aha, D. W., Kibler, D. & Albert, M. K. Instance-based learning algorithms. *Mach. Learn.* **6,** 37–66; https://doi.org/10.1007/BF00153759 (1991).

7. Breiman, L. Random Forests. *Mach. Learn.* **45,** 5–32; https://doi.org/10.1023/A:1010933404324 (2001).
